# Cancer immunotherapy: nanodelivery approaches for immune cell targeting and tracking

**DOI:** 10.3389/fchem.2014.00105

**Published:** 2014-11-26

**Authors:** João Conniot, Joana M. Silva, Joana G. Fernandes, Liana C. Silva, Rogério Gaspar, Steve Brocchini, Helena F. Florindo, Teresa S. Barata

**Affiliations:** ^1^Faculdade de Farmácia, Instituto de Investigação do Medicamento (iMed.ULisboa), Universidade de LisboaLisboa, Portugal; ^2^EPSRC Centre for Innovative Manufacturing in Emergent Macromolecular Therapies, UCL School of PharmacyLondon, UK

**Keywords:** nanosystems, cancer, targeted delivery, cell tracking, immunotherapy

## Abstract

Cancer is one of the most common diseases afflicting people globally. New therapeutic approaches are needed due to the complexity of cancer as a disease. Many current treatments are very toxic and have modest efficacy at best. Increased understanding of tumor biology and immunology has allowed the development of specific immunotherapies with minimal toxicity. It is important to highlight the performance of monoclonal antibodies, immune adjuvants, vaccines and cell-based treatments. Although these approaches have shown varying degrees of clinical efficacy, they illustrate the potential to develop new strategies. Targeted immunotherapy is being explored to overcome the heterogeneity of malignant cells and the immune suppression induced by both the tumor and its microenvironment. Nanodelivery strategies seek to minimize systemic exposure to target therapy to malignant tissue and cells. Intracellular penetration has been examined through the use of functionalized particulates. These nano-particulate associated medicines are being developed for use in imaging, diagnostics and cancer targeting. Although nano-particulates are inherently complex medicines, the ability to confer, at least in principle, different types of functionality allows for the plausible consideration these nanodelivery strategies can be exploited for use as combination medicines. The development of targeted nanodelivery systems in which therapeutic and imaging agents are merged into a single platform is an attractive strategy. Currently, several nanoplatform-based formulations, such as polymeric nanoparticles, micelles, liposomes and dendrimers are in preclinical and clinical stages of development. Herein, nanodelivery strategies presently investigated for cancer immunotherapy, cancer targeting mechanisms and nanocarrier functionalization methods will be described. We also intend to discuss the emerging nano-based approaches suitable to be used as imaging techniques and as cancer treatment options.

## Introduction

Cancer is a heterogeneous disease that results from a multi-step process, characterized by uncontrolled tumor cell proliferation, invasion and metastasis. Tumor cells have also the ability to evade cell death (Fernald and Kurokawa, 2013) and to escape immune system surveillance (Zitvogel et al., 2006).

Despite improvements in diagnosis and therapies, cancer is still the most fatal disease worldwide with 11.5 million deaths being predicted in 2030. Strategies for cancer treatment include chemotherapy, radiotherapy, immunotherapy and surgery (Wu et al., 2014). Many of these approaches are unspecific with severe side effects (Peer et al., 2007). More effective and specific alternative treatments continue to be needed. In fact, it has been described that those single treatment regimens have limited chances to eliminate cancer cells in a permanent manner due to its heterogeneous nature (Hanahan and Weinberg, 2000; Helmy et al., 2013). The success of cancer therapy is dependent on the development of additional strategies to overcome severe side effects, drug resistance and circumvent tumor evasion mechanisms (Girardi et al., 2001; Dunn et al., 2002; Koebel et al., 2007; Chen et al., 2014b; Xu et al., 2014).

Although the general body immune response is often not robust enough to escape to cancer cell tactics (Palucka and Banchereau, 2012), our understanding of tumor immunology has been evolving. It is accepted that tumor cells, parts of tumor cells or even specific substances isolated from tumor cells can be recognized by the immune system, which can then respond to these malignant cells. The possibility for immune system-based responses has brought new insights into the development of novel cancer immunotherapy treatments. Immunotherapy has begun to meet its promise for cancer treatment. Monoclonal antibodies (mAbs) to specific targets that are engaged with tumor mechanisms are used clinically, including alemtuzumab (lymphocytic leukemia) and trastuzumab (breast cancer) (Kirkwood et al., 2012). Additionally, cancer vaccination has shown encouraging preclinical results and has also been extensively explored, being mostly directed to the destruction of tumors by strengthening the immune system (Rosenberg, 2001; Palucka and Banchereau, 2012).

The recognition of the crucial role of T-lymphocytes in cancer for immune-mediated treatments has contributed to the exhaustive characterization of tumor-associated antigens (TAAs). Of particular interest are the cytotoxic T Lymphocytes (CTL), which upon antigen recognition can selectively target and destroy malignant cells presenting epitopes which have been recognized. However, their isolated response is often not enough and the development of an optimal cancer vaccine seems to be dependent on an effective stimulation and cooperation between CTL and T helper (Th) cells specific for a tumor epitope (Fong and Engleman, 2000; Banchereau et al., 2001; Palucka and Banchereau, 2012).

In addition to the evolution of tumor immunology, there has been progress in the development of nanodelivery systems. These systems have the potential to overcome some of the drawbacks of current chemotherapy and radiotherapy therapies. As reviewed by Chow and Ho (2013), nanosystems can display improved pharmacokinetics and targeting of tissues and cells to enhance efficacy, specificity and lower toxicity. Accordingly, nanosystems designed to target immune molecules and cells may allow the development of approaches that will use the patient's immune system as a more specific tool to fight cancer.

Nano-based platforms have also been explored for immune cell labeling, using fluorescence and molecular imaging techniques. As a result, immune cell mechanisms engaged in cancer development and tumor metastasis can thus be better understood, guiding the development of advanced platforms able to specifically target and track immune cells.

## Cancer and the immune system

### Innate and adaptive immunity to cancer

The immune system is composed of two main branches—innate and adaptive immune responses. The innate immunity is a non-specific first line defense of our body against antigens. It comprises anatomic, physiologic, phagocytic, and inflammatory barriers, such as skin or macrophages and neutrophils. On the other hand, adaptive immunity is a highly specific component of the immune system, which is stimulated by a specific antigen challenge to the organism. Still, the latter is not independent from the innate response, since antigen-presenting cells (APCs), involved in innate immunity, play a pivotal role in specific immunity activation (Roitt and Delves, 2001; Kindt et al., 2006).

Dendritic cells (DCs), along with macrophages and B lymphocytes, are described as APCs (Roitt and Delves, 2001; Gogolak et al., 2003; Kindt et al., 2006). DCs are the most powerful “professional” APCs, being present in the majority of mammalian tissues and acting as an interface between innate and adaptive immunity. They control and regulate the immune system. DCs are organized in an intricate network throughout the human lymphatic and non-lymphatic tissues, having different functions, depending on their stage of maturation (Banchereau et al., 2003; Bodey et al., 2004; Palucka and Banchereau, 2012). Non-activated immature DCs capture antigens and induce tolerance in the steady state, whereas mature antigen-loaded DCs can prime an antigen-specific immune response. DCs can also be categorized in three main subsets—(i) Langerhans cells (LCs); (ii) interstitial DCs (intDCs) and (iii) plasmocytoid DCs (pDCs). Though all subsets derive from the same precursor cells—CD34+ hematopoietic stem cells, found in the marrow—they are originated from two major distinct pathways (Banchereau et al., 2003). LCs and intDCs arise from the myeloid pathway, are CD11c+ and both produce IL-2. LCs are present in stratified epithelia, like skin and upper airways, whilst intDCs may be found in all other tissues. Additionally, intDCs can secrete IL-10 and elicit naïve B cell differentiation (Gogolak et al., 2003). The other parallel pathway originates phenotypically CD11c− pDCs with the ability to produce high amounts of type I interferon and to modulate T cell differentiation (Gogolak et al., 2003).

In tumor immunology, DCs are crucial for the presentation of TAAs and to stimulate the immune system after DC activation (Palucka and Banchereau, 2012). DCs patrol the different tissues, processing exogenous and endogenous antigens that are then presented to T lymphocytes, after DC maturation. The maturation process of DCs can be induced directly through “danger signals” detected by pattern recognition receptors (PRRs) or triggered by the presence of inflammatory mediators, such as TNF-α or IL-1β (Bodey et al., 2004).

Antigen presentation to T lymphocytes by DCs occurs through T-cell receptors (TCRs) that recognize antigens bound to major histocompatibility complex (MHC) molecules. MHC proteins can be sorted in two main groups: MHC class I—expressed on the membrane of the majority of nucleated cells in vertebrates—and MHC class II, only found in APCs (Levine and Chain, 1991; Bodey et al., 2004). After the contact of a naïve T cell with MHC-antigen complex, T cells proliferate and differentiate in both memory T cells and effector T cells. Effector T cells may be divided in T helper (Th—CD4+) or T cytotoxic (Tc—CD8+) cells (Guermonprez et al., 2002; Gogolak et al., 2003). The stimulation of Tc cells can lead to the generation of CTLs that secrete low levels of cytokines, unlike Tc cells. However, they display cell-killing action, controlling and eliminating cells that exhibit any type of antigen, such as infected cells or tumor cells (Gogolak et al., 2003). Other innate lymphocytes subsets, such as γδ T cells, natural killer (NK) and natural killer T (NKT) cells, have been reported as being engaged in a complex immunomodulatory network, displaying anti-tumor activity. Preclinical studies described that NKT cells can exhibit anti-tumor or immune-regulatory mechanisms (Gajewski et al., 2013).

The interaction among B cells, T cells and mature DCs results in an integrated immune response. Therefore, DC migration from the tumor site of antigen capture to secondary lymphoid organs can thus greatly broaden antigen-specific T cell responses, promoting effective anti-tumor immune responses that will lead to tumor rejection and regression (Palucka and Banchereau, 2012).

A promising nano-based strategy has been designed in order to develop synthetic DCs for T cell activation and immunotherapy, based on semi-flexible and filamentous polymers (Mandal et al., 2013). Effective antitumor-immune responses are thus dependent on the development of alternative systems to deliver antigens to DCs and promote their presentation to T cells. These factors are important to bear in mind when developing an effective vaccine (Gajewski et al., 2013).

### Cancer immune regulation and evasion mechanisms

#### Cancer immunosurveillance

Paul Ehrlich proposed the concept of the immune system as a useful strategy against cancer, in the beginning of the Twentieth century (Ehrlich, 1909). Some decades after, Thomas and Burnet postulated the immunosurveillance theory based on Ehrlich's hypothesis. Cancer arousal was suggested to be caused by the lack of efficiency of the immune system or the modification in antigen expression of tumor cells, leading to its evasion (Burnet, 1957; Thomas, 1982). Thomas and Burnet also claimed that antitumor immune response generally happens at an early stage of the cancer development (Burnet, 1957; Thomas, 1982). Therefore, once the tumor has grown, it escaped the immunosurveillance barrier and started developing additional mechanisms to evade from the immune system (Ahmad et al., 2004). Nevertheless, Strutman's later studies showed that cancer susceptibility of immune-competent mice was similar to that observed in mice with major immunodeficiency, setting against the immunosurveillance hypothesis (Shankaran et al., 2001; Dunn et al., 2002). In the beginning of this century, the immunosurveillance hypothesis was revised, as several studies have shown that the immune system may not only destroy tumor cells but also shape their phenotypes, leading to reduction of immunogenicity (Shankaran et al., 2001; Dunn et al., 2002; Schreiber et al., 2011).

Currently, there is increasing evidence that tumor cells can be recognized and destroyed by the immune system, as developing tumor cells often co-express tumor antigens and ligands for activating receptors (Schreiber et al., 2011). Therefore, it is important to describe which immune components display major roles in tumor rejection. It is also important to clarify the appropriate time and efficient type of action (Swann and Smyth, 2007).

#### Cancer immunoediting and cancer-related inflammation

As reviewed by Schreiber et al. (2011), cancer immunoediting can be divided in three different phases: “elimination,” “equilibrium” and “escape.”

In the first stage—“elimination”—both innate and adaptive immunities act combined to identify the formation of tumor cells and to destroy them, resembling the immunosurveillance theory. Although many mechanisms are still poorly known, it has been reported that cytokines, “danger signals” and DCs have important roles in this phase (Sims et al., 2010; Vesely et al., 2011). It has also been suggested that the required components for an effective “elimination” depend on specific characteristics of the tumor cells, such as its origin or anatomical location (Sims et al., 2010). If the “elimination” stage is well succeeded, tumor cells are destroyed, constituting an endpoint for cancer immunoediting (Schreiber et al., 2011; Vesely et al., 2011).

The next stage—“equilibrium”—is described as a period of tumor latency. In other words, when a tumor cell survives the elimination phase, the adaptive immune response can control tumor cell growth and shape its immunogenicity. “Equilibrium” is believed to be the longest phase of cancer immunoediting process. It seems to allow cancer cells to reside in patients' body even decades before it restarts to grow and become clinically evident (Schreiber et al., 2011; Vesely et al., 2011).

The third phase—“escape”—occurs when tumor cells have developed the ability to evade the mechanisms of recognition of the immune system and/or their elimination. Tumor cells are thought to progress from “equilibrium” phase to “escape” through several mechanisms and/or pathways. For instance, an alteration in immune system response, which may be triggered by cancer-induced immunosuppression or a change in tumor cells induced by immunoediting, or even immune system deterioration (Schreiber et al., 2011; Vesely et al., 2011).

#### Cancer immune evasion mechanisms

Cellular immunity has been shown to play a major role in the control of tumor generation. Even though, recent findings revealed that tumors often manage to evade it through several different mechanisms. It has been reported that there is a reduction or even loss of MHCI molecules, mostly associated to gene mutations or impairment of MHCI-dependent antigen processing (Garrido and Algarra, 2001; Ahmad et al., 2004; Vesely et al., 2011). In addition, an antigenic drift in cancer cells has lately been observed and appears to be related with the mutation, loss or down-expression of TAAs in tumor cells (Uyttenhove et al., 1997; Ahmad et al., 2004). Similarly, the lack or reduction of the expression of co-stimulatory patterns by tumor cells direct T lymphocytes to an anergy state. These mechanisms altogether seem to reduce and difficult the detection of cancer cells by CTLs and NK cells, which consequently leads to tumor growth (Ahmad et al., 2004).

Alterations in apoptotic receptor signaling seem to help tumor cells to evade the immune system. Molecules such as phosphatidylinositol 3-kinase (PI3K), protein kinase B and Fas ligand (FasL) have modified expression and might be implicated in this process (Davidson et al., 1998; Osaki et al., 2004).

Tumor eradication is also dependent on the manipulation of immunosuppressive properties of tumor microenvironment, where inducing and suppressing cytokine imbalance impairs DC activation and maturation, compromising immune cell effector properties and supporting tumor growth. Tumor cells can indeed secrete immunosuppressive molecules, including vascular endothelial growth factor (VEGF), IL-10 and transforming growth factor-β (TGF-β) (Fortis et al., 1996; Tsushima et al., 1996; Oyama et al., 1998). VEGF appears to be responsible for down-regulation of NF-κB expression, which interferes in DC maturation and differentiation, limiting the immune response against tumor cells (Oyama et al., 1998). On the other hand, TGF-β1 is an immune suppressive cytokine involved in the conversion of CD4+T cells into immunosuppressive T regulatory (Treg) cells that are mainly produced by DCs and tumor cells (Zou, 2005).

These immunosuppressive molecules are interesting targets to achieve tumor growth inhibition and might be a very useful tool for cancer immunotherapy. The use of nanoparticles (NPs) containing small interfering RNA (siRNA) to knock-down TGF-β in the tumor microenvironment has resulted in increased levels of CD8+ T cells and lower number of Treg cells, leading to tumor growth inhibition by 52% (Xu et al., 2014). A similar strategy using polyethylenimine-capped silica NPs carrying VEGF siRNA has been designed as a highly effective approach for lung cancer growth suppression and metastasis (Chen et al., 2014b). High levels of indoleamine 2,3-dioxygenase have also been found in tumor microenvironment, reducing tryptophan pool levels, which drive T lymphocytes to be arrested at G1 phase of the cell cycle (Ahmad et al., 2004).

### Tumor microenvironment: tumor-inflitrating immune cells and related regulatory pathways

The progress of cancer disease results from several mechanisms developed by tumors to evade antitumor immune responses (Section Cancer immune evasion mechanisms), which has been associated mostly to tumor microenvironment molecular pathways and infiltrating cells at this particular region, rather than the ignorance and defects of anti-tumor T cells (Gajewski et al., 2013; Ma et al., 2013). In fact, the presence of different cells and their dynamic interaction with malignant cells have a profound effect on tumor progression (Mishra et al., 2010; Bussolati et al., 2011; Cortez-Retamozo et al., 2012; Rahir and Moser, 2012).

It is widely accepted that the density of T cell infiltrates within tumor microenvironment is the most important factor to predict cancer patients' survival (Eerola et al., 2000; Oble et al., 2009; Mahmoud et al., 2011). Nevertheless, macrophages are also currently recognized as a fundamental cell type. As a heterogeneous population, its dual function toward cancer is determined by their polarization status (Mantovani and Sica, 2010). Macrophages are regulated by transcription factors, which will lead to different phenotypes of tumor-associated macrophages (TAMs). M1 and M2 have been already characterized, being associated to the pathogenesis of several diseases, namely inflammatory and tumor diseases (Sica et al., 2006). Indeed, pro-inflammatory M1 macrophages, after being activated by IFN-γ, favor Th1 immune cell activity and potentiate the eradication of malignant cells. On the other hand, M2 phenotype enables Th2 immune responses and regulate tissue repair, presenting pro-tumoral abilities in several tumor types (Sica and Mantovani, 2012; Cornelissen et al., 2014). Moreover, the production of several cytokines, such as IL-1, IL-6, IL-10, VEGF, and TGF-β by M2 TAMs elicits the proliferation and metastasis of tumor cells (Biswas and Mantovani, 2010). As a result, it has been described that the number of M2 macrophages and the overall M2/M1 ratio of TAMs are important predictors of survival for distinct types of cancers, namely melanoma (Erdag et al., 2012; Herwig et al., 2013), ovarian cancer (Lan et al., 2013; Colvin, 2014), T-cell (Niino et al., 2010), and B-cell lymphomas (Nam et al., 2014), breast (Leek et al., 1996) and pancreatic cancer (Ino et al., 2013).

It is important to mention however that the M1 and M2 classification of TAMs is not static, being usually very complex and seems to be dictated by several mediators resultant from cellular cross-talk and environmental conditions (Cai et al., 2012; Escribese et al., 2012; Shime et al., 2012). Even tough, the causes underlying the differentiation of TAMs to M1 or M2 phenotypes are not yet fully understood. Type I interferon pathway seems to be fundamental for the activation of innate immune response against tumor cells. However, the production of type I interferon by DCs remain an underexplored issue (Fuertes et al., 2011).

DCs are also present within tumor microenvironment, where they can recognize and capture live and dying tumor cells (Dhodapkar et al., 2008; Ma et al., 2013). Their presence in tumors of different stages and grades correlates to prolonged disease survival and lower invasiveness, as reviewed in Palucka and Banchereau (2012). Even though, some of this heterogeneous hematopoietic lineage displays anti-tumor effects while others present immunosuppressive functions at tumor site. Actually, tumor-infiltrating DCs functionality may vary according to the combination of environmental factors and pathways within variable tumor site. Among DC subsets, it should be emphasized the role of tumor-infiltrating plasmacytoid DCs (pDCs) and CD8α+ DCs lineage, being the first often related to T cell tolerance, while the latter is in fact particularly efficient in the cross-presentation of antigens via MHCI pathways and thus in cytotoxic T-cell immunity (Hildner et al., 2008; Fuertes et al., 2011; Watkins et al., 2011).

The characterization of different solid tumors, as melanoma, showed the presence of tumor-infiltrating lymphoid cell lineage, including CD8+ T cells. Their function is mainly compromised by immune system-inhibitory pathways at tumor microenvironment, enabling T cell anergy (Gajewski et al., 2013). It has been reported the presence of high amounts of CD4+ Foxp3+ regulatory T cells (Treg cells) that are attracted by the chemokine CCL22 via CCR4 (Toulza et al., 2010; Spranger et al., 2013). However, the function of T-cell subsets within tumor microenvironment is highly complex, depending on several factors, such as the type of receptors primed.

Another hypothesis for the presence of T cells within the tumor microenvironment of certain tumors may be related to the formation of a lymph node-like structure called tertiary lymphoid tissues, where it is possible to find B cells, T cells and activated DCs (Messina et al., 2012). Still, it is not clear if the formation of those lymphoid structures is involved in tumor growth *in vivo*. On the other hand, tumor-infiltrated T cells can express CCL21. CCL21 is related to tumor tolerance by stimulating naïve T cells to which the presentation of TAAs will not be efficient due to the absence of co-stimulatory factors (Shields et al., 2010).

NK, NKT and γδT cells also seem to have an important role in the immunomodulation of tumor microenvironment (Peng et al., 2007; Mishra et al., 2010; Marcu-Malina et al., 2011; Liu et al., 2012). The antitumor effect of NK has been linked to solid and hematopoietic tumors, while γδT cells and NKT cells have been involved in tumor inhibition. However, they show immunoregulatory functions in certain circumstances that are not completely known (Peng et al., 2007; Mishra et al., 2010; Marcu-Malina et al., 2011; Liu et al., 2012). A promising strategy has been focused in the stimulation of DCs by α-galactosylceramide to prime NKT, promoting the production of IFN-γ (Shimizu et al., 2013).

Besides these cells, tumor stroma has also been associated with tumor growth and includes different elements as collagen, endothelial cells, fibroblast and several macrophage subsets, which contributes for tumor immune evasion. In addition, higher levels of angiogenic factors were found in tumors where the presence of tumor-infiltrating T cells is poor (Danhier et al., 2010). The major immunosuppressive mechanisms include the secretion of IL-10, TGF-β, and CCL22 by M2 macrophages (Condeelis and Pollard, 2006). The trafficking of T cells within tumor microenvironment has been related to the secretion of different chemokines by stromal cells, namely CXCL9 and CXCL10 (Gooden et al., 2011).

Nevertheless, the inherent complexity of the immune regulation within tumor microenvironment and the incomplete definition of those multiple mechanisms demand additional efforts to characterize these processes. Such characterization would support the development of translational alternative immunotherapies (Mellman et al., 2011). For example, the presence of tumor-infiltrating T cells within tumor site may indicate that this particular type of tumor is a potential candidate for an immunotherapeutic strategy due to their ability to support the migration of T cells toward this particular region. However, the multiple factors involved in the immune system inhibition indicate that the use of complementary targeted strategies to improve the presence of anti-tumor T cells and the knock-down of immune inhibitory pathways may lead to optimal therapeutic approaches.

Combinatory approaches for cancer therapy need indeed to consider the successful modulation of the tumor-associated cytokine network and cell communication within tumor microenvironment. This will prevent the inhibition of anti-tumor responses and down-regulate the proliferation of malignant cells. The characterization of these immunoregulatory processes and the deeper understanding of the immunological features within tumor microenvironment have fostered the recognition of biomarkers. Such recognition has been driving the design of novel targeted therapies to block those pathways, including targeted nanomedicines to tumor microenvironment to better avoid off-targeted effects. The anti-CTL4 monoclonal antibody ipilumimab approved in 2011 by the US Food and Drug Administration (FDA) to treat patients with advanced melanoma, constitutes the first successful approach that targets one of those inhibitory pathways (Mellman et al., 2011).

The design of these tumor-targeted systems is also influenced by a variety of specific features presented by this region, when compared to healthy tissues. Among those different properties, vasculature and pH have been the most explored toward the development of alternative and specific therapeutic nanosystems (Fernald and Kurokawa, 2013; Torchilin, 2011). Angiogenesis guarantees the supply of oxygen and different nutrients to tumor cells. It results from the action of different factors, as pro-angiogenic proteins, extracellular matrix proteins and matrix metalloproteinases. This process is fundamental for the progression of the disease and has guided the development of different targeted nanocarriers due to the particular morphology of the blood vessels, as reviewed by Torchilin (2011). In fact, abnormal architecture of blood vessel caused by incomplete angiogenesis allows the retention of different nanodelivery systems specifically at this particular tumor region, due to the so-called “Enhanced Permeability and Retention” (EPR), which will be described in Section Passive Targeting.

## Cancer immunotherapeutic interventions

Cancer immunotherapy has been explored for some decades. This term is often used to describe treatments based on modulation of the immune system through “active” or “passive” approaches. The concept of immunotherapy relies on specific immune mechanisms and targets, which could confer greater efficacy and specificity with less toxicity. Therefore, improving the presence of anti-tumor T cells and the knock-down of immune inhibitory pathways, leading to optimal therapeutic approaches.

### Active cancer immunotherapy

Active cancer immunotherapy or cancer vaccination consists in direct stimulation of the patient's immune system so it can act against tumor cells. Unlike infectious disease vaccination, which efficiency is based mainly on neutralizing antibodies and B-lymphocyte response, cancer vaccination depends on the induction of CTL responses and on the administration of TAAs to stimulate a systemic immune response.

Cancer vaccines are expected to induce a tumor specific immune response able to either eliminate the malignant cells or keep it under constant restraint, delaying tumor recurrence and prolonging survival. Both prophylactic and therapeutic vaccine-based cancer therapies have been proposed to enhance a specific immune response to tumor cells, concerning DC activity, as summarized in Vacchelli et al. (2012). It has also been reported the prominence of DCs on CTL induction, thus becoming a striking target for cancer vaccination (Section Strategies for DC Targeting).

The extensive research has led to engineered biotech molecules, such as proteins, peptides, antibodies and oligonucleotides, designed to enhance immune-based mechanisms, being promising players to re-shape the future of immunotherapeutic outcomes. However, as these candidates move toward clinical investigation, it becomes clear that their biological effect depends on the development of a tool able to attain their transport across biological barriers. Accordingly, the potential of these bioactive molecules has pointed nanomedicines as an approach to ensure the target selectivity and safety required for their therapeutic *in situ* efficacy, enabling their clinical application.

As discussed by Silva et al. (2013), an ideal vaccination strategy involves the administration of the most immunogenic TAAs along with the most effective adjuvants, including delivery platforms. This will prime the tumor- specific T cells, induce tumor-specific antibodies and kill tumor cells by host immune effector mechanisms.

Several TAAs have been identified and characterized permitting their use in the design of targeted delivery systems (Bos et al., 2012; Engels et al., 2013). TAAs can be sorted as shared tumor antigens—when present in many types of tumors and with a distinct or absent expression on normal tissues (i.e., MAGE, GAGE and NY-ESO1)- or unique tumor antigens. These antigens result from point mutations or splicing alterations and are expressed only by a specific tumor (Higgins et al., 2009; Pejawar-Gaddy et al., 2010). However, those newly identified antigens, as recombinant proteins, are usually weakly immunogenic, requiring multiple administrations and their association with adjuvants. It has been described that both antigen and adjuvant must act in a concerted way on the same APC, which can be provided by a singular delivery system (Schlosser et al., 2008; Krishnamachari et al., 2011; Raaijmakers et al., 2013).

As previously mentioned, the focus of cancer vaccines is the stimulation of a cell-mediated immunity, rather than humoral responses. As many TAAs are intracellular proteins, fragments of these peptides must be presented on the cell surface bound to MHC class I molecules to be recognized by the immune system (Henderson et al., 2005). Indeed, after the recognition of TAA-MHCI complexes, in lymph nodes (Manolova et al., 2008), CD8+ T lymphocytes can proliferate and differentiate into CTLs. CTLs are then able to migrate to peripheral tissues to develop contact-mediated cytotoxicity activity and secrete effector cytokines as IFN-γ and TNF-α, leading to local inflammation (Ahlers and Belyakov, 2010).

Pattern recognition receptors, mainly the toll-like receptor (TLR) family, are suitable targets to potentiate the presentation of TAAs through MHCI pathway to CD8+ T cells and increase cancer immunotherapy efficacy. Among TLR agonists, both cytosine phosphorothioate-guanine motifs (CpG; TLR9-ligand), double stranded RNA mimic polyinosinic:polycytidylic acid (poly(I:C); TLR3-ligand) and monophosphoryl lipid A (MPL) have been associated to stronger anti-tumor immune responses (Banchereau et al., 2003; Hildner et al., 2008; Radford and Caminschi, 2013).

Generally, TAAs and TLR ligands carried by polymeric particles have the ability to escape the degradation in endosomes and reach the cytosol in higher concentrations than those administered in soluble form. Those antigens can thus be presented by MHC-I molecules more effectively and for longer periods of time, leading to an effective cellular response, which is fundamental for a successful eradication of cancer cells.

### Passive cancer immunotherapy

Passive immunotherapy is based on the administration of *ex vivo* generated immune effector molecules or cells, such as antibodies and CTLs, respectively. These molecules or cells can target specific receptors, leading to enhanced efficacy of the treatment and also to fewer side effects.

#### Monoclonal antibodies (mAbs)

Monoclonal antibodies are the main cancer immunotherapy used currently in clinic to treat solid tumors and lymphomas (Krishnamachari et al., 2011). For example, trastuzumab has been used to treat HER2^+^ breast cancer and adenocarcinoma, whilst alemtuzumab has been applied in chronic lymphocytic leukemia treatment (Lee et al., 2013).

The mechanism of action of mAbs is related to their ability to interfere with both growth factor ligands and receptors or pro-apoptotic targets, inducing apoptosis of cancer cells. Besides, mAbs may activate components of the immune system through Fc-region-based mechanisms. This leads to antibody-dependent cell-mediated cytotoxicity (ADCC) and complement-dependent cytotoxicity (CDC) responses by macrophages and NK cells (Krishnamachari et al., 2011).

The use of mAbs in clinic has been increasing in the last decades. The first generation of mAbs used in cancer therapy was originated from mouse. Their origin often resulted in limited half-life, decreasing mAbs efficacy. Further progresses conducted to the development of chimeric mAbs, with enhanced properties, and then humanized mAbs. Nowadays, fully human mAbs are already available (Lee et al., 2013). Several novel mAbs for different cancer types are presently in clinical trials, as reviewed by Lee et al. (2013). For example, ganitumab—for pancreatic cancer –and necitumumab—for non-small cell lung cancer—are now in phase III of clinical trials.

#### Adoptive T-cell therapy

This approach is based on the transfer of mature tumor-reactive T lymphocytes to act against tumor cells. Unlike cancer vaccines, this strategy is independent from an immune response elicited by an exogenous antigen. Instead, it relies on the delivery of a great amount of *ex vivo*-expanded cells (Gajewski, 2012; Kirkwood et al., 2012; Helmy et al., 2013).

Adoptive T-cell therapy with tumor-infiltrating lymphocytes (TILs) has been proposed. In a successful study, autologous TILs—T cells with potent antitumor activity found within tumors—were harvested, activated *ex vivo* and reinfused in patients. The total remission was reported in more than 20% of the treated patients (Rosenberg et al., 2011).

Complementary research has been made to improve T-cell adoptive therapies. Genetically engineered T cells are under study, in order to manipulate the properties of the administered T-cell population, such as proliferation and migration characteristics (Liu and Rosenberg, 2001; Hinrichs et al., 2011). Also, T cells have been genetically modified to have antitumor specificity by introducing a T-cell receptor for a particular tumor, as previously described in a review by Helmy et al. (2013).

## Delivery strategies for immune cell targeting and tracking

### Strategies for DC targeting

Since the role of DCs in inducing CTL immunity is well established, several studies have been made in order to use DC-based cancer vaccines in tumor immunotherapy.

#### Ex vivo

These vaccines use isolated CD14+ monocytes or CD34+ DC precursors from an individual. After being isolated, these cells are then cultured and differentiated in immature DCs (Romani et al., 1994; Chapuis et al., 1997). The following process is TAA-loading of DCs, which consists in adding proteins, peptides or tumor lysates to its culture medium or through transfection. Additional maturation stimuli, such as CD40L or pro-inflammatory cytokines, may be used to ensure DCs will be able to induce a strong cellular immune response. Finally, loaded mature DCs are administered back into the patient by intravenous (i.v.), subcutaneous (s.c.), intradermal (i.d.), intratumoral (i.t.) or intralymphatic (i.l.) route (Hamdy et al., 2011).

The use of a tumor cell to stimulate DCs seems to induce a better immune response, but it is limited by a possible induction of autoimmune diseases, due to the lack of antigen specificity among the undefined antigen found at cancer cell surface.

Whichever the type of antigen used to pulse DCs, although it has been reported that this approach is safe and able to induce CTL immunity, the clinical observed goal is low, possibly due to the *in vivo* general complex interactions between immune cells (Rosenberg et al., 2004).

DC therapy involves the isolation, culture and stimulation of patient's monocytes and macrophages *ex vivo* using TAAs (Cho et al., 2011). When administered back to the patient, antigen-loaded DCs will bypass the *in vivo* uptake of tumor antigens. DCs are already activated and therefore they are able to migrate to the secondary lymph nodes wherein they will trigger T cells. However, the relative short half-life of TAA-MHC complexes on DC membrane surface, and the low percentage (3–5%) of DCs that can migrate to the lymph nodes and contact with T cells can contribute to the low rate of success of these vaccines (De Vries et al., 2003; Hamdy et al., 2011). Also, being produced specifically for a particular patient, *ex vivo* DC-based vaccines are a highly complex, laborious, time-consuming and expensive approach. Futhermore, the vaccine quality might depend on the clinic where it is produced, once there are several variable parameters in the process, such as dose of DCs and posology (Hamdy et al., 2011). The type of DCs stimulated, antigen loading method and DC maturation level are also important aspects to be characterized to better understand the adjuvant role of DCs.

#### In vivo

**Figure 1:**
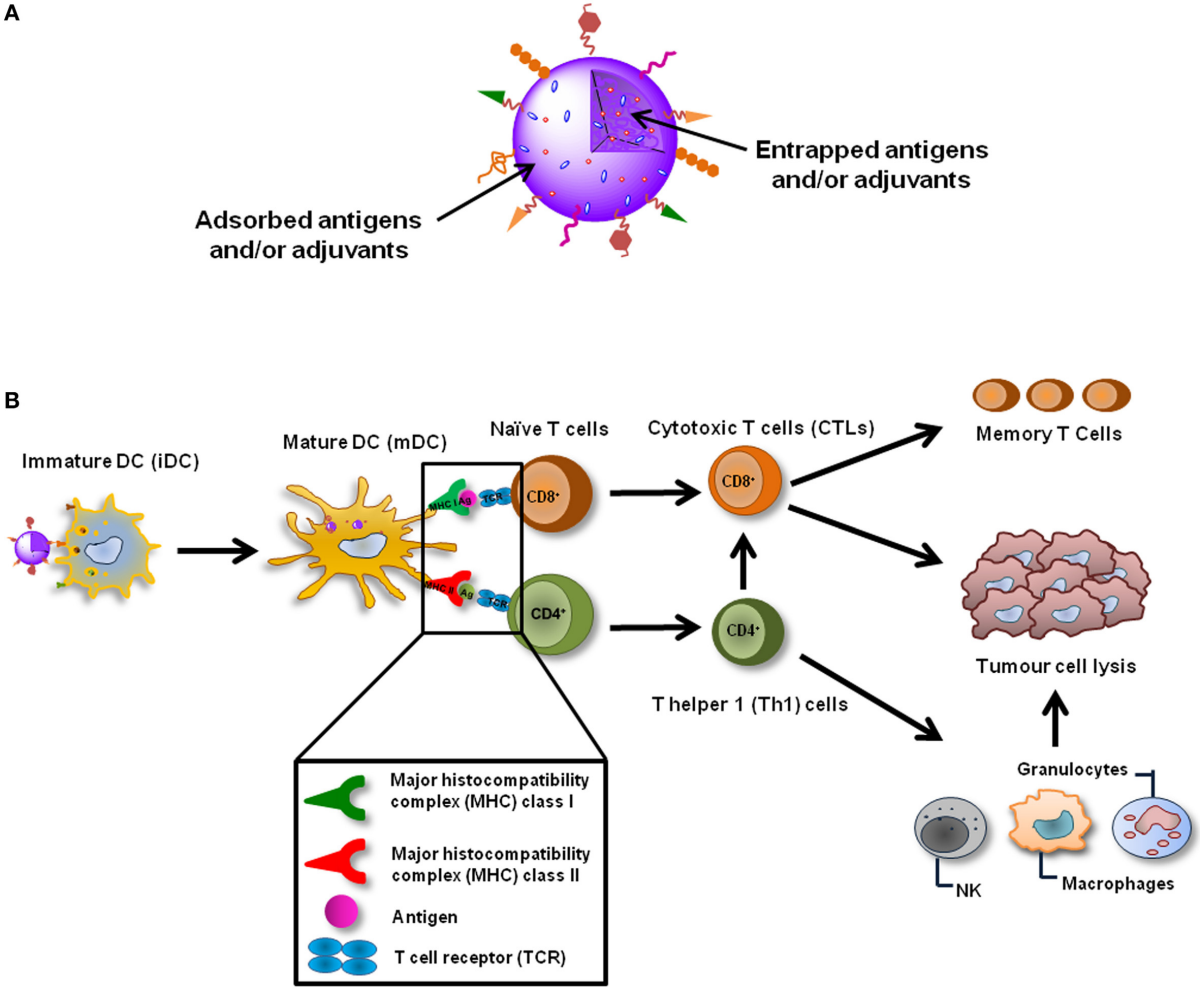
**Nanoparticulate cancer vaccines. (A)** NPs are able to deliver several TAAs and adjuvants simultaneously, enabling a coordinated activation of DCs. NPs can also be functionalized in order to actively target DCs *in vivo*, increase their cellular internalization and immunogenicity or even target specific intracellular compartments. **(B)** NP-based cancer vaccines can be targeted to DCs *in vivo* and after their internalization induce the maturation of these cells. TAAs and adjuvants are simultaneously released inside the same DC which guaranties its coordinated activation. TAAs are presented trough MHC class I and class II molecules to CD8+ and CD4+ naïve T cells which recognize the processed antigens through TCRs. Activated CD8+ T cells differentiate into CTLs, which can destroy tumor cells, and memory T cells, that are important to avoid recidivism and metastasis. CD4+ T cells should differentiate in Th1 cells, which will potentiate the action of CTLs and will also activate cells of the innate immune system, such as NK cells, granulocytes and macrophages that play a role in the tumor destruction process as well.

To overcome the lack of clinical efficacy of *ex vivo* DC-based cancer vaccines, it is extremely recommended to develop an alternative way to target antigens directly to DCs *in vivo*, which can be achieved using peptide-based vaccines. These are mainly based on MHCI peptides, which are simple to produce and administer, and guarantee DC activation and expansion for prolonged periods of time (Figure 1) (Cheong et al., 2010; Silva et al., 2013). However, the cytoplasmic delivery of the antigen is limited by low membrane permeability and frequent destruction after intracellular entry, being their immunogenicity considerably lower than the traditional vaccines. Hence, their association to potent adjuvants, as particulate vaccine delivery systems, or immunomodulatory molecules is being widely investigated (Al-Hanbali et al., 2006; Hillaireau and Couvreur, 2009; Sharp et al., 2009; Shahar et al., 2010; Smith et al., 2012).

Numerous studies have demonstrated that these delivery platforms could increase the uptake of antigens and adjuvants by DCs, leading to better immune responses (Diwan et al., 2002; Schlosser et al., 2008; Florindo et al., 2009a). *In vivo* DC-targeted vaccines are able to deliver, within the same platform, both antigens and additional stimuli (i.e., adjuvants) to the same cell in its natural environment, enhancing and maximizing the outcome (Kazzaz et al., 2006). Particulate delivery systems range from micro and nanoparticles, liposomes, to virus-like particles (VLPs). Unlike *ex vivo* DC vaccines, the clinical intervention is limited to vaccine administration, sparing time in fastidious cycles of blood withdrawal and *in vitro* cell culture. Also, it offers on-shelf products, which can be produced at large scale with cost reduction and increased quality.

### Nanotechnology-based approaches as immune cell targeted delivery systems

Nano-based systems have been described as platforms for targeting and delivery of not only therapeutic agents, but also nanodevices and analytical systems for theranostics. The range of applications of nanosystems can include drug delivery, cancer and gene therapy, as well as imaging and cell tracking through biomarkers and biosensors (Rawat et al., 2006) (Supplementary Material). Nanosystems have been used to increase the resolution of clinical imaging, with improved sensitivity and specificity, leading to earlier diagnostics and real-time results. This may allow the use of prophylactic measures, to avoid the progress of the disease or to greater efficacy of therapies, due to an earlier treatment (Riehemann et al., 2009).

The development of nano-based systems has provided protection strategies for incorporated agents, such as biomolecules—nucleic acids, peptides and proteins—which are generally quickly degraded when administered *in vivo*. Therapeutic agents can be embedded, encapsulated, or even adsorbed or conjugated onto the nanosystems, which can be modified and associated to other adjuvants to achieve an optimized release profile (Mahapatro and Singh, 2011). Usual concerns about the administration of these biomolecules have been eased, since lower doses are generally used and a more restricted distribution is achieved (Rawat et al., 2006). In fact, the widely recognized versatility of nanotechnology strategies allows the accurate design of multifunctional nanocarriers. These, in turn, can be functionalized by ligands of different natures to promote a targeted delivery of their cargo both at cellular and subcellular level.

Nanocarriers can also potentiate the cytosolic delivery of biomolecules as siRNA and miRNA, important gene expression regulators, providing their escape from endo-lysosomal compartments. miRNAs are short oligonucleotides (18–22 nucleotides) and are involved in multiple pathways related to the development and differentiation of cells, and in the pathogenesis of cancer, constituting a valuable target Chen et al., 2014b; Gajos-Michniewicz et al., 2014). However, its *in vivo* application demands the development of cell-specific delivery approaches to promote their biological effect, which are currently underexplored.

**Figure 2:**
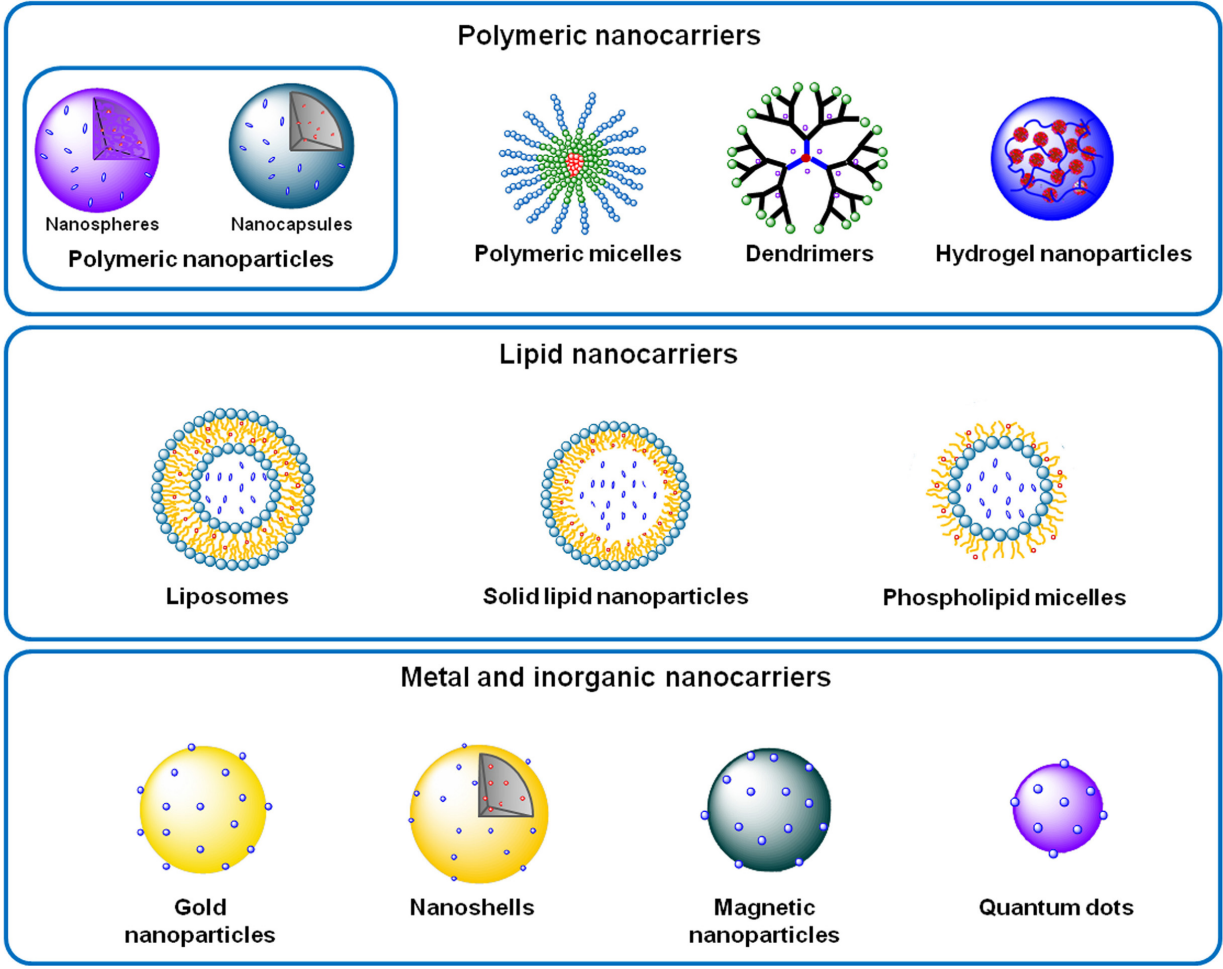
**Examples of polymeric, lipid, and metal and inorganic nanocarriers**.

The modulation and regulation of the pathophysiology dynamics at the molecular level has enabled nanomedicines to achieve a disease control with an unprecedented precision. Therefore, several nano-based systems composed by diverse materials, and thus presenting different characteristics, have been proposed and sorted in polymeric, lipid, metal and inorganic nanocarriers (Figure 2). Among them, it is important to underline liposomes, polymeric nanoparticles and micelles and dendrimers.

Besides the strong demand to develop alternative therapeutic options to address unmet clinical needs, the novel nanotechnology-based platforms have although important challenges, not only for industry but also for government agencies. Efficacy and safety are evaluated on proof-of-concept studies, but the manufacturing process must be robust by identifying all its critical points and thus implementing “quality-by-design” (QbD) concept or improved process analytical technologies (PAT).

#### Liposomes

**Figure 3:**
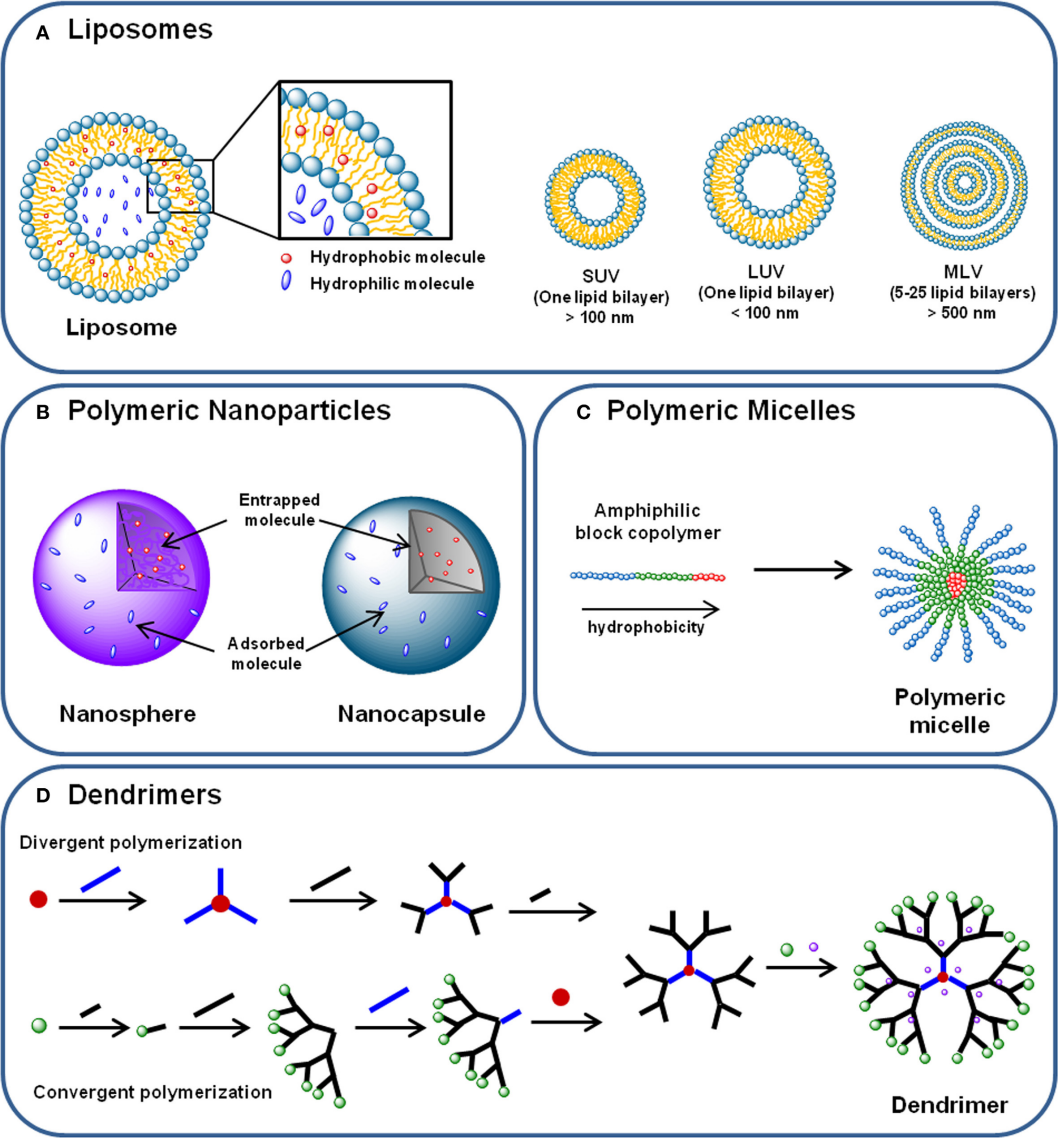
**(A)** Liposomes are phospholipid and cholesterol self-assembled bilayer membranes that enclose an aqueous core, where hydrophilic molecules can be incorporated. Hydrophobic compounds can also be incorporated in the lipid bilayer. Liposomes can be classified in (i) small unilamellar vesicles (SUVs); (ii) large unilamellar vesicles (LUVs) and (iii) multilamellar vesicles (MLVs), according to their size and lamellarity. **(B)** Polymeric nanoparticles are submicron spherical entities composed by a polymeric compact net than can either constitute a polymeric matrix—in the case of nanospheres—or a polymeric wall surrounding a vesicular core—nanocapsules. Nanoparticles can transport hydrophilic and hydrophobic molecules either entrapped in the polymeric matrix or core, or adsorbed to their surface. **(C)** Polymeric micelles are self-assembled spherical nanocarriers formed by amphiphilic block copolymers. In aqueous medium, the block copolymers arrange themselves in a disposition where the most hydrophobic parts of their chains form a hydrophobic core—where hydrophobic molecules can be incorporated –, and the most hydrophilic regions of the polymer chain are displayed outoward. **(D)** Dendrimers are hyperbranched nanocarriers formed by a central core, branching monomers and functionalized peripheral groups. Dendrimer synthesis can start from the core element (divergent polymerization) or from the peripheral branching units (convergent polymerization), resulting in a structure with a hydrophilic surface and a hydrophobic central core. Molecules can be transported by dendrimers either incorporated in the core and branches, either conjugated to the terminal groups.

Liposomes consist of self-assembled lipid bilayer membranes with size ranging from 90 to 150 nm, which are formed by phospholipids and cholesterol that enclose an aqueous core (Figure 3A). Phospholipids are composed by hydrophilic heads and hydrophobic long tails. Thus, as previously described in several reviews, their structure allows hydrophilic molecules to be incorporated within the inner compartments, while the hydrophobic compounds will be entrapped within the hydrophobic bilayer (Sahoo and Labhasetwar, 2003; Aslan et al., 2013; Sharma et al., 2013).

The potential use of liposomes as delivery systems is based on the fact that they provide a slow and sustained release, improving the accumulation of the entrapped molecules. Also, on their ability to decrease cytotoxicity of incorporated molecules, since they modulate the biodistribution and pharmacokinetics (Khan et al., 2008; Sharma et al., 2013). Having in consideration their biocompatibility, the biodegradability and ability to cross lipid bilayers and cell membranes, liposomes have been proposed as delivery platforms for vaccines, anticancer drugs and gene therapy (Ewert et al., 2005). However, one of the major drawbacks of conventional liposomes is the short circulation time, being rapidly removed by mononuclear phagocytes of the reticular endothelial system (RES). Stealth liposomes, or long-circulating liposomes, have been developed to overcome this problem. They consist in liposomes that are sterically stabilized, presenting thus a prolonged half-life (Frank, 1993; Krishnamachari et al., 2011).

Regarding the success attained by liposomal platforms in the clinic and advanced-stage clinical trials, several liposomal-based delivery systems are nowadays offered as an anticancer strategy, such as liposomal doxorubicin, cytarabine and cisplatin (Abraham et al., 2005; Huwyler et al., 2008; Aslan et al., 2013). The use of liposomes for doxorubicin delivery prevents the damage of heart and renal healthy tissues that is usually induced by the extreme toxicity of the drug (Abraham et al., 2005). Moreover, doxorubicin has already been formulated in active targeted liposomes for breast cancer therapy, using engineered peptide ligands (Sharma et al., 2013). Other attractive approach is the use of liposomes as carriers for antisense oligonucleotides, as siRNA, in cancer therapy (Tari et al., 1996).

Van Broekhoven et al. (2004) have reported a DC-targeting vaccine, based on a liposomal formulation, as an outstanding platform to induce a highly effective immunity against tumor cells (Van Broekhoven et al., 2004). Preclinical studies of liposome-DNA complexes have also been described, constituting an effective strategy to elicit anti-tumor immunity (U'ren et al., 2006).

The phase I clinical trial of a liposomal cancer vaccine for breast, ovarian and prostate cancer has already been reported. It has been proved that this peptide vaccine, which is intended to elicit multi-functional T-cell responses, is safe and immunogenic (Berinstein et al., 2012).

Additionally, liposomes have been studied as carriers for alternative bioorganic and biodegradable contrast agents, as glycogen and poly-L-Lysine. With these liposomes, it was possible to develop an *in vivo* multi-color magnetic resonance imaging for lymph node mapping (Chan et al., 2014).

#### Polymeric nanoparticles (NPs)

Polymeric NPs are submicron-sized polymeric colloidal particles with excellent features as vehicle for the delivery of drugs, biomolecules and genes (Panyam and Labhasetwar, 2003; Mahapatro and Singh, 2011).

Polymer properties such as biocompatibility, low toxicity and biodegradability have highlighted polymeric NPs as an interesting delivery strategy. The chemical structure of the polymers is easily modified, allowing the development of multifunctional engineered systems. Nanoparticle size, shape and surface properties can also be tailored, as well as the degradation kinetics and mechanical properties (Albertsson, 2002).

Polymeric NPs are usually highly stable and can easily entrap and/or adsorb both hydrophilic and hydrophobic molecules with good efficacy (Gelperina et al., 2005). The drug entrapment protects molecules from degradation (Singh and Lillard, 2009). Additionally, as nano-sized polymeric particles, these carriers are easily transported through extra and intracellular barriers. As a result, entrapped agents may be delivered site-specifically, for instance in inflamed areas or tumors, after crossing the endothelium (Prokop and Davidson, 2008; Singh and Lillard, 2009).

Two different types of polymeric NPs are usually considered: nanospheres and nanocapsules (Figure 3B). Nanospheres consist in a polymeric matrix in which the drug or cargo is homogenously dispersed, whereas nanocapsules are vesicular systems formed by a polymer wall that surrounds a core containing the cargo (Singh and Lillard, 2009).

Several methods have been used to produce polymeric nanoparticles. Some of the most studied are spray-drying, salting out, nanoprecipitation and emulsion-based methods, The latter, in particular, lies on an emulsification process with the removal of organic solvents used for polymer dissolution, by extraction or evaporation. The emulsified organic drops containing the polymer originate nanoparticles, when the organic solvent is eliminated (Lassalle and Ferreira, 2007).

Nevertheless, it is important to bear in mind that the chosen method will influence the characteristics of the obtained NPs, such as the size and the surface. Besides, it is crucial to have a great knowledge about the different experimental variables, in order to achieve the intended formulation characteristics (Gorner et al., 1999; Lassalle and Ferreira, 2007).

A large number of polymers from different origins have already been described as useful materials for polymeric NP production and used in preclinical studies. Polymers can be from natural origin, as chitosan, or synthesized, as polylactic acid and poly-lactic-co-glycolic acid (PLGA) (Krishnamachari et al., 2011; Mizrahy and Peer, 2012). Particulate adjuvants, such as PLGA and PCL NPs, have generated a lot of interest due to their biodegradability, biocompatibility and mechanical strength. (Danhier et al., 2012) has nicely reviewed the main properties and applications of PLGA-based nanocarriers. These NPs can also act as adjuvants, maintaining the antigenicity and immunogenicity of encapsulated proteins. In fact, PLGA, used for decades in humans, is the most studied polymer for vaccine formulation and it was shown to increase antibody and cellular responses to antigen-loaded PLGA NP (Johansen et al., 2000; Shen et al., 2006; Chen et al., 2014a). PCL has a great potential for developing antigen controlled release matrices by its low degradation rate, hydrophobicity, good drug permeability, *in vitro* stability and low toxicity. The adjuvant effect of PCL NPs to induce immune responses against an infectious disease was previously confirmed by several studies (Benoit et al., 1999; Florindo et al., 2008, 2009b; Labet and Thielemans, 2009). If the encapsulated antigen fails to induce DC activation, these NPs can be modified with maturation signals at their surface for direct ligand-receptor interaction, as mannose receptor is overexpressed at DCs and macrophage cell surface. Chitosan NPs, for instance, are an interesting strategy for gene delivery, namely small interfering RNA (siRNA). As chitosan is positively charged, electrostatic interactions occur with negatively charged siRNA, and thus the biomolecule is safely carried to its *in vivo* target (Aslan et al., 2013).

Nanocarriers produced using polypeptide-based polyanionic, zwitteronic and polycationic polymers (e.g., polyglutamic acid, polyarginine) have also been described (Christian et al., 2009). These are endosomolytic polymers and have been used to promote the cytosolic delivery of these biomolecules. Although clinical trials with peptide-based cancer nanovaccines have shown little success, more recent research has been developed to improve them, using novel polymeric NPs systems.

It has been reported that PLGA NPs loaded with melanoma antigens can elicit effective anti-tumor activity by CTLs *in vivo* (Zhang et al., 2011; Ma et al., 2012). DC-targeting chitosan NPs, carrying IL-12, were also used in a preclinical study. The administration of this nanovaccine in an animal model resulted in suppression of tumor growth and increased induction of apoptosis (Kim et al., 2006).

Regarding immune cell tracking, biodegradable PLGA NPs have been used in a combined multimodal imaging strategy for a DC-targeting nanovaccine. Superparamagnetic iron oxide particles and a fluorescently labeled antigens were incorporated within the same nanosystem, allowing not only the analysis and quantification of NPs uptake, but also the subcellular tracking of NPs (Cruz et al., 2011).

#### Polymeric micelles

Polymeric micelles are self-assembled spherical nanocarriers formed by amphiphilic block copolymers in aqueous medium (Figure 3C). A hydrophobic core and a hydrophilic surface compose these structures, and their size ranges from 10 to 100 nm (Torchilin, 2001; Jhaveri and Torchilin, 2014).

Polymer micelles have been investigated as delivery systems for poorly water-soluble/hydrophobic drugs due to the hydrophobic core. It has been shown that micelles can enhance the bioavailability of hydrophobic molecules, which is reassured because they protect the drug from *in vivo* degradation (Torchilin, 2001; Jhaveri and Torchilin, 2014). Other advantages of polymeric micelles are the low toxicity, the prolonged circulation time and good levels of accumulation in tumor areas (Ganta et al., 2008). In an experiment with nude mice xenograft model, PLGA-PEG polymeric micelles have shown increased tumoral uptake (Yoo and Park, 2004).

Novel pH-responsive polymer micelles formed by an N-(2-hydroxypropyl) methacrylamide corona and a propylacrylic acid (PAA)/dimethylaminoethyl methacrylate (DMAEMA)/butyl methacrylate (BMA) core have already been investigated for antigen trafficking modulation in DCs. The results showed that this nanosystem facilitates the antigen delivery to DCs in the lymph nodes and enhances CD8+ T cell responses, being thus a potential carrier for cancer vaccines (Keller et al., 2014). Also, micelles formed by DMAEMA and pyridyl disulfide ethyl methacrylate (PDSEMA), carrying both CpG ODN and protein antigens, have shown to elicit and increase cellular and humoral immune response by modulating and stimulating antigen cross-presentation, as summarized by Wilson et al. (2013).

#### Dendrimers

Dendrimers consist in hyperbranched spherical nanocarriers formed by a central core, branching monomers and functionalized peripheral groups. Dendrimers can be produced by convergent or divergent polymerization of branching units, resulting in a structure with a hydrophilic surface and a hydrophobic central core (Figure 3D) (Lee et al., 2005). Their main physicochemical features are low viscosity, hyperbranched molecular topology, marcromolecular size, high density of chemical functionality and multiple end groups that can be chemically functionalized (Lee et al., 2005). Also, the depolymerization of dendrimers can be tailored in order to control the release profile of the loaded agents, as described in a review by Wong et al. (2012). Besides vaccines, therapeutic and targeting carriers, dendrimers have also been reported as diagnostic tools due to their ability to protect imaging agents, decreasing its toxicity and enhancing specificity (Yang et al., 2009).

Nowadays, the most described family of dendrimers is the well-studied polyamidoamine (PAMAM). Poly(propyleneimine) and peptide dendrimers, such as poly(L-glutamic acid) dendrimers, have also been studied (Nanjwade et al., 2009). Linear poly(glutamic acid) is a poly(amino acid) polymer with considerable potential for antigen delivery to DCs, and adjuvant properties for DC maturation, able to induce CTLs (Yoshikawa et al., 2008). Additionally, it has been shown to be safe for use in clinic (Chipman et al., 2006) providing the necessary safety profile for human use. These glycopeptide dendrimers have shown promise for antitumor and antiviral prophylactic or therapeutic vaccines, as well as antiviral agents (Niederhafner et al., 2008). Several formulations have reached clinical trials as vaccines against breast (Gilewski et al., 2007), prostate (Slovin et al., 2003), and small cell lung cancers (Krug et al., 2004) with encouraging results. Even though, further investigation must be done in order to guarantee the long-term safety, before they become clinically available (Aslan et al., 2013).

### Influence of nano-based technology properties in cellular uptake

Arguably, the weakest link in preclinical experimentation of nanodelivery systems is the continued failure to document dynamic processes (over time) using complex biosystems as models, i.e., a systems biology approach. The outcome of different classes of nanomedicines under preclinical and clinical evaluation has demonstrated that their main biological consequences of cellular or subcellular targeting and access are closely related to materials intrinsic properties (Ehmann et al., 2013).

The uptake of TAAs, carried within nano-platforms, by DCs is in fact influenced by several particulate physicochemical properties. Size, shape, surface charge, hydrophobicity and receptor interactions are generally underlined (Foged et al., 2005; Bachmann and Jennings, 2010). Particulate vaccines, such as whole-cell vaccines, virosomes, VLPs or formulated delivery platforms such as liposomes, micro and NPs have great surfaces with electrostatic or receptor-interacting properties, leading to an increased interaction when compared to soluble antigens (Bachmann and Jennings, 2010). Also, it has been reported that particulate size can direct the DC subset target. However, the ideal dimensions of NPs for APC uptake are still under discussion. In fact, small size platforms (<200 nm) may drain freely to LNs, being thus taken up by LN-resident DC subsets such as CD8α+, which seems an advantage for cancer immunotherapeutic approaches. However, delivery systems greater than 200 nm appear to be taken up by circulant monocytes, which differentiate after particle uptake and migrate to LNs afterwards (Manolova et al., 2008). According to Foged and colleagues, NP size should be 0.5 μm or less to be quickly and efficiently incorporated by DCs (Foged et al., 2005).

#### Size

NP size appears to influence the cellular uptake mechanism and the endocytic pathway of NPs, dictating their ultimate intracellular fate and thus overall biological effect. NPs may be assimilated by receptor-mediated endocytosis, clathrin-dependent and/or caveolae-mediated, and phagocytosis, or through a receptor independent mechanism—macropinocytosis. Particulate systems with a larger diameter (>0.5 μm) tend to be assimilated through macropinocytosis and/or phagocytosis by some specific cells, as macrophages and Langerhans cells in the skin. Smaller particles usually enter the cell through endocytosis. NPs with size <150 nm are generally taken by cells via classic receptor-mediated endocytosis (clathrin-dependent) or endocytosis caveolae-mediated if ranging from 50 to 80 nm (Pelkmans and Helenius, 2002). These NPs with size equivalent to viruses are usually able to initiate a virus-like immune response with activation of CTL and Th1. On the other hand, larger particles normally generate a similar immune response to that induced by bacteria, with Th2 activation and antibody production (Xiang et al., 2006).

#### Shape

Besides size, it has also been reported that particle shape may influence cellular uptake and biodistribution. Although it has been suggested non-spherical particles may be valuable for their increased blood circulation time, due to reduced phagocytosis by unspecific cells, they also demonstrated decreased cellular uptake, when compared to spherical NPs. According to Gratton et al., rod-shaped NPs show the highest uptake performance, followed by spheres, cylinders and finally cubical NPs (Gratton et al., 2008).

#### Surface charge

NP surface charge also seems to play an important role in their particle internalization and thus will also determine the nature of the induced immune response (Xiang et al., 2006). As cell membrane charge is negative, positively charged molecules/systems will show high affinity to it. After cellular uptake, it has been observed that negatively charged or neutral NPs tend to localize within lysosomes, whilst positively charged NPs showed ability to escape from these. Cationic NPs were found in the perinuclear area and have been reported as effective for uptake by macrophages and DCs (Thiele et al., 2003; Yue et al., 2011). On the other hand, the interaction of those delivery systems with cell depends on multiple factors and some studies have reported the presence of neutral NP at endoplasmic reticulum, suggesting their ability to escape degradation at lysossomal/endossomal compartment (Zhou et al., 2014).

### Nanocarriers for tumor and immune cell targeting

#### Passive targeting

Passive targeting results from the transport of nano-based systems across the abnormal leaky vasculature of tumors, into the tumor interstitium or cells, by their movement within fluids—convection—or by passive diffusion. Whereas convection is observed for larger molecules, compounds with low molecular weight cross the membranes by diffusion, depending only on the concentration gradient (Iyer et al., 2006; Danhier et al., 2010).

As blood vessels architecture and its regulation are compromised, caused by unpaired angiogenesis, nanocarriers tend to accumulate selectively in tumor interstitium due to the “Enhanced Permeability and Retention (EPR) effect.” The increased size of gaps in endothelial cells creates pores ranging from 10 to 1000 nm, which along with the poor lymphatic drainage, contributes to the EPR effect, that was first described by Matsumura and Maeda (1986); Yuan et al. (1995); Danhier et al. (2010). This effect has become very important for the design of targeted nanocarriers for cancer therapies. It has been reported that NP levels of accumulation in tumor interstitium are 10–50-fold higher than in normal tissues, leading to improved therapeutic efficacy and less side effects (Iyer et al., 2006; Danhier et al., 2010).

#### Active targeting

Nanotechnology-based strategies have been explored as platforms for drug delivery, cancer vaccination and/or diagnosis, due to their capacity for overcoming biological barriers and to modulate payloads' intracellular trafficking. These nanoparticulate systems present a good potential for site-selective delivery by binding recognition ligands to NP surface, which can enhance NP endocytosis, influencing their intracellular trafficking and thus inducing prolonged effects (Danhier et al., 2010).

**Figure 4:**
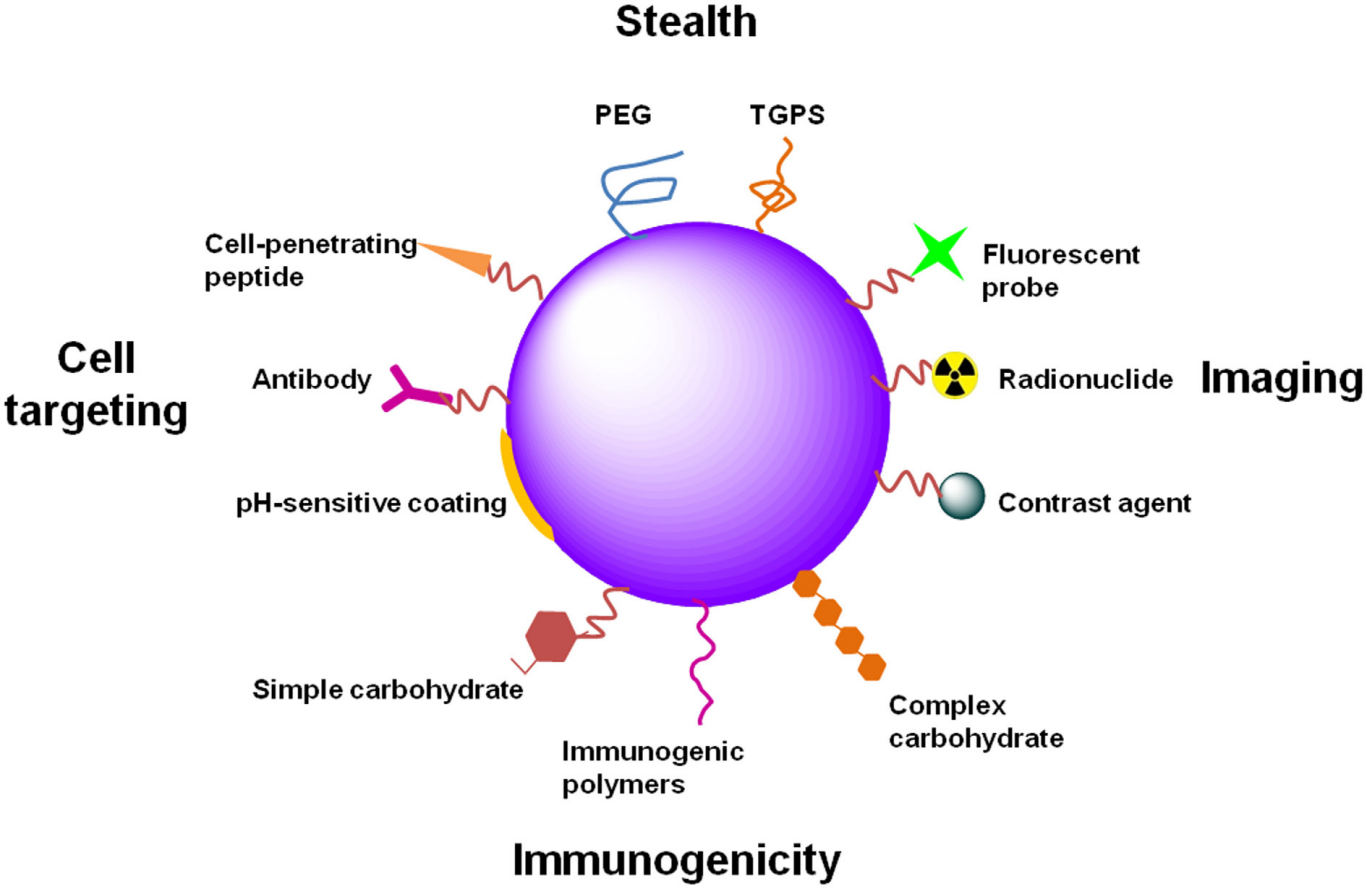
**Examples of NP functionalization**. NPs can be functionalized differently in order to attain distinct goals. PEG or TGPS functionalization provide stealth properties to NPs, avoiding capture by phagocytic cells and increasing their circulation time. Functionalization of NPs with imaging agents, such as fluorescent probes, radionuclides or contrast agents (e.g., gold or magnetic NPs), provide applicability of NPs to diagnostic, theranostic or even *in vivo* real-time imaging. The immunogenicity of NPs can be increased for immunotherapy or prophylactic vaccination. Different molecules can be used for that propose, such as PAMPs (several carbohydrates, lipids or nucleic acids) or immunogenic polymers (e.g., chitosan, alginate, poloxamers). Specific tissue and cell targeting can be achieved through the functionalization of NPs with antibodies directed to specific or overexpressed antigens. Cell-penetrating peptides can improve NP internalization. pH-sensitive coatings allow drug release in specific tissues or intracellular compartments in a pH-dependent manner.

Surface functionalization of nano-based systems (Figure 4) has been used to improve tissue and cell surface antigen targeting, thus moderating non-specific distribution and prolonging the blood circulation time of nano-based systems (Alexis et al., 2008).

**Figure 5:**
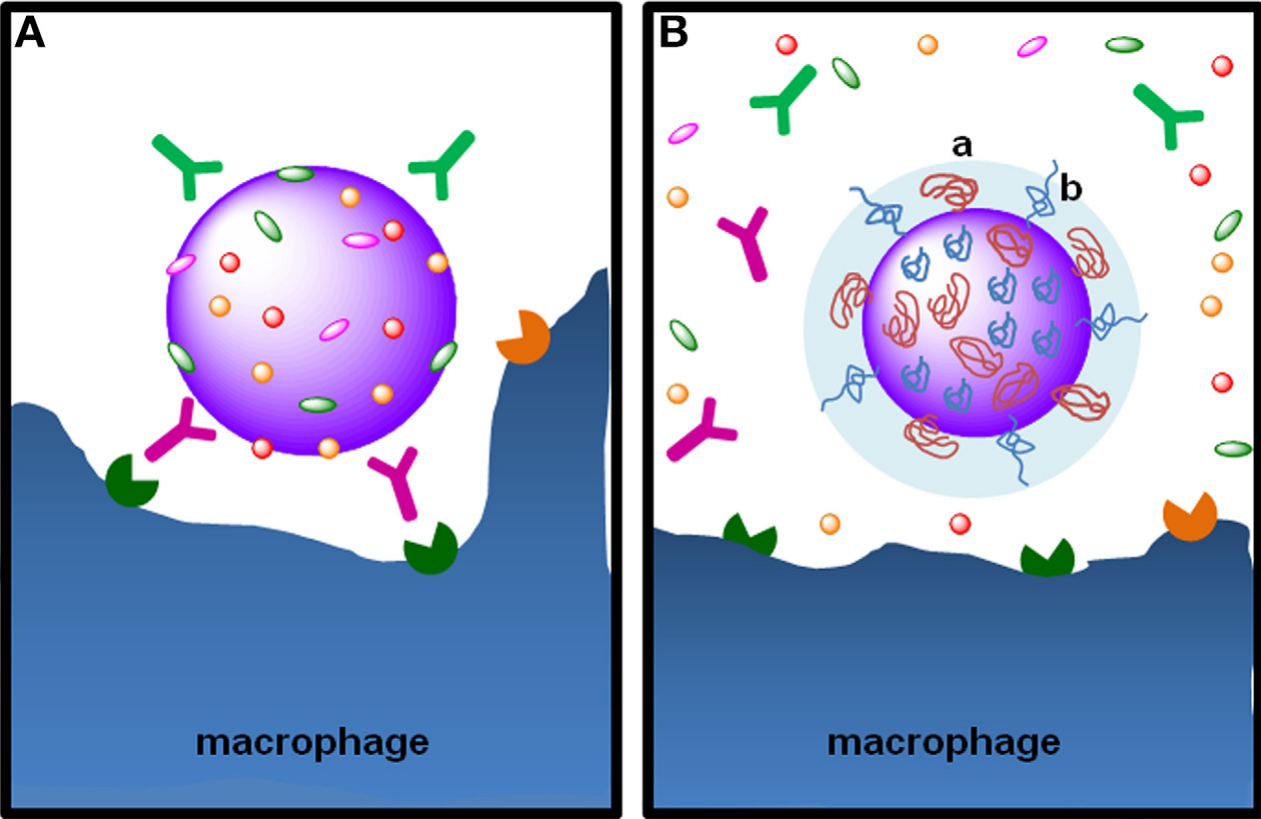
**The stealth effect from NP functionalization with PEG. (A)** Particulate foreign entities in body fluids are promptly covered with opsonins, such as the immunoglobulins IgG and IgA and the complement proteins C3b C4b, in a process called opsonization. Opsonins mark the particulate entity to phagocytosis through their recognition by Fc receptors on phagocytic cells, such as macrophages. **(B)** Functionalization of NPs with PEG by grafting, conjugation or adsorption—note the “mushroom-like” (a) or “brush-like” (b) configuration of PEG chains—provides steric stabilization and stealth properties, preventing the adsorption of opsonins at the surface of nanoparticles. PEG hydrophilicity attracts water molecules to particle surface avoiding the adsorption of opsonins at NP surface, rendering them “invisible” to phagocytic cells.

PEGylation is a widespread strategy to improve the half-life time of nanocarriers, through steric stabilization and “stealth” properties. It relies on the introduction of poly(ethylene glycol) (PEG) molecules by conjugation, grafting or adsorption onto the surface of nanosystems (Figure 5). The terminal groups of PEG chains also present very suitable moieties to attach functional ligands and attain active-targeted carriers (Freichels et al., 2012). The conjugation of antibody fragments to PEG ends, using disulfide bonds, may consist in an interesting strategy to develop platforms for active targeting (Brocchini et al., 2008). D-α-tocopheryl polyethylene glycol succinate (TPGS) has been reported as an alternative to PEG (Pan and Feng, 2008).

**Figure 6:**
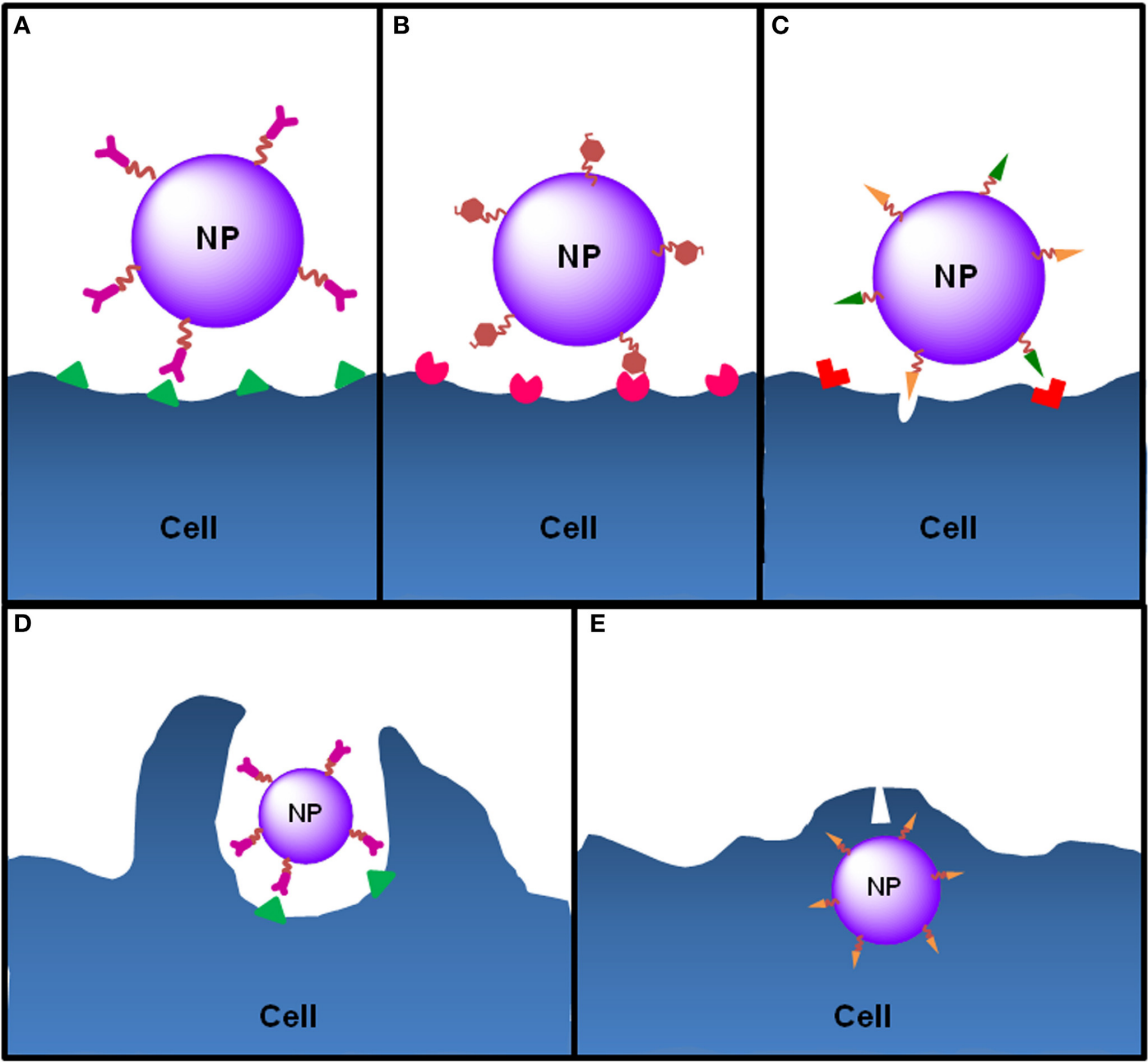
**Ligand-cell interaction and NP internalization**. NPs can be functionalized with different ligands to increase cell targeting and NP internalization. **(A)** Functionalization of NPs with antibodies allows the targeting of antigens exclusively expressed or overexpressed by target cells (e.g., anti-CD205 antibody to target CD205 on DCs or anti-HER2 antibody to target HER2 on breast cancer cells). **(B)** In order to target DCs, NPs can be functionalized with molecules that mimic PAMPs, normally carbohydrates, nucleic acids or lipids, which are recognized by PRRs expressed by DCs. For instance, mannose or fucose residues are recognized by the mannose receptors—a C-lectin receptor. Bacterial lipopolysaccharide or flagellin target TLR4 and TLR5 on DCs, respectively. **(C)** Cell-penetrating peptides are small amino acid sequences normally used by viruses or bacteria to facilitate cellular invasion by those pathogens and can be used to increase the internalization of NPs. Functionalized NPs see their internalization by target cells increased essentially by two mechanisms: induction of endocytosis upon ligand-receptor binding, which happens to NPs functionalized with ligands such as antibodies, PAMPs or some penetrating peptides that induce receptor-mediated endocytosis (e.g., integrins) or **(D)** through direct cell penetration across the plasma membrane (e.g., antimicrobial peptides or histidine-rich peptides) **(E)** or both (e.g., HIV TAT peptide).

Active-targeted nanosystems are based on the design of nanocarriers with bioactive ligands placed onto their surface or periphery. They will be recognized by overexpressed molecular patterns at the tissues/cells intended to target, facilitating NP recognition and subsequent receptor-mediated endocytosis (Figure 6) (Cheng et al., 2007; Kumar et al., 2009; Danhier et al., 2010; Aslan et al., 2013; Nicolas et al., 2013; Wang et al., 2013a; Gao et al., 2014). Surface modifications represent an outstanding tool for cell targeting allowing a specific contact of nanoparticulate systems with critical immune cells, as evidenced in Stephan et al. (2010). For example, the ligand DEC-205 is highly expressed by CD8+DCs, cells particularly efficient at “cross-presenting” exogenous antigens on MHCI, constituting a highly relevant pathway for the development of a cytolytic immune response. Moreover, recent studies have indicated that the triggering of CD40 on APCs can lead to CD8 T-cell effectors, without the need of common stimulation by MHCII-related Th cells via CD40 ligands (Vonderheide et al., 2013). Mannose receptors at DCs are also associated to ligand internalization and further processing and presentation by immune cells, leading to a more extensive immune response (Lu et al., 2007; Carrillo-Conde et al., 2011; Silva et al., 2013).

These ligands, such as peptides, antibodies and antibody fragments, carbohydrates and even vitamins, may be either attached before the nanocarrier production or afterwards. Liking ligands prior to nanocarrier production may be advantageous, so that the conjugation yield of the ligand to the polymer can be assessed and controlled. Nanocarriers can be thus produced with a well-characterized (co)polymer and the density of ligands on their surface can be tailored. Physicochemical properties of the polymers must be evaluated after ligand conjugation, because the hydrophilic/hydrophobic balance may be altered, particularly if macromolecules are linked (Betancourt et al., 2009; Sperling and Parak, 2010; Nicolas et al., 2013).

The strategy of attaching ligand molecules after nanocarrier production is usually applied, when antibodies, proteins and polypeptides are chosen as targeting agents. As some organic solvents are generally used in the preparation of nanosystems, this method is preferred to avoid denaturation of the secondary structure of the ligands. Also, since they are bulky molecules, they will disturb the hydrophilic/hydrophobic balance which can difficult the method of nanocarrier production (Nicolas et al., 2013). The drawbacks of this approach are related with subsequent purification of the formulation and its characterization. The processes frequently used for purification, such as centrifugation, filtration and dialysis, may degrade or alter the nanosystems. Additionally, it is usually difficult to prove that the ligand is covalently linked to the surface of the nanocarrier and not only adsorbed (Nicolas et al., 2013).

***Ligation strategies for functionalization***. Several pathways have been developed to attach ligands onto nanosystems surface, such as the carbodiimide strategy, the Michael addition pathway, the biotin–streptavidin approach and the Copper-catalized ligation method (Betancourt et al., 2009; Nicolas et al., 2013). The native terminal groups of some polymers or specific moieties, introduced through chemical modifications, are generally used to apply these schemes of functionalization. For instance, carboxylic acid terminals in aliphatic polyesters and poly(ethylene glycol) (Betancourt et al., 2009).

The most used scheme is based on the carbodiimide chemistry. It relies on the coupling of a molecule containing a terminal amine group with another with an N-hydroxysuccinimide (NHS) ester end or an end group that can be easily esterified to NHS moiety (Betancourt et al., 2009; Nicolas et al., 2013). The Michael addition pathway is based on the thiol-maleimide coupling. Maleimide-polymers are used to produce nanocarriers, which are then decorated with thiol-containing targeting agents (Betancourt et al., 2009; Nicolas et al., 2013). However, the presence of native thiol groups in some molecules, as proteins and peptides, is usually low (or absent in some cases) and many are hard to access. To overcome this, disulfide bonds can be reduced in thiol groups or heterobifunctional cross-linking agents may be used (Nicolas et al., 2013). The biotin–streptavidin approach utilizes a strong non-covalent biological interaction between biotin and avidin (Betancourt et al., 2009; Nicolas et al., 2013). Still, for this strategy, a targeting agent is usually chemically bound to avidin, which is a bulky glycoprotein that may then obstruct the interaction ligand-receptor, essential for targeting (Betancourt et al., 2009). The Copper-catalized ligation is a highly efficient method, based on a cycloaddition reaction that fits in the “click chemistry” class of reactions. The chemical reaction is developed in mild conditions and with little or absent byproducts. The major disadvantage of this approach is the elimination of the Cu-based catalyst used for the reaction (Nicolas et al., 2013).

***Functionalization of nanosystems for immune cell targeting***. Extensive research has been made regarding cell surface receptors in immune cells, the so-called PRRs. PRRs recognize pathogen-associated molecular patterns (PAMPS) and are involved in several stages of the immune response, from its initiation and proliferation, to its execution (Kumar et al., 2009). Different types of molecules may act as PAMPs, known as “danger signals,” for instance lipids, lipoproteins, proteins, carbohydrates and nucleic acids. The recognition of PAMPs by PRRs triggers immune responses by activating multifactorial signaling pathways. This leads to the induction of inflammatory responses mediated by several cytokines and chemokines (Kumar et al., 2009).

Several classes of PRRs have been reviewed, such as TLRs, retinoic-acid inducible gene (RIG)-like receptors (RLRs), nucleotide oligomerization domain (NOD)-like receptors (NLRs), DNA receptors (cytosolic sensors for DNA), scavenger receptors, and C-type lectin receptors (CLRs) (Kumar et al., 2009; Carrillo-Conde et al., 2011; Shen et al., 2013; Silva et al., 2013). In mammals, the most studied PRR class is the TLRs class. TLRs are predominantly expressed by APCs, as DCs, but they are also found on cells of the adaptive immune system, such as in αβT cells, regulatory T cells, and γδT cells, as well as NKT cells (Wesch et al., 2011). Through TLR activation, both the innate and the adaptive immune responses can be engaged, either by direct activation of TLRs with their ligands on T and B cells, or by indirect mechanisms involving TLR-activated DCs (Silva et al., 2013). C-type lectin receptors (CLRs) belong to another class of PRRs expressed by APCs. This receptor family is characterized by the presence of domains that bind to carbohydrates (Van Kooyk, 2008). CLRs are specific receptors particularly engaged in the internalization of antigens. CLRs enable the intracellular uptake and processing of antigens, as well as influence their cytosolic fate and the loading on MHC class I and II (Unger and Van Kooyk, 2011).

Regarding the involvement of PRRs in several strategic immune pathways, the design of nano-based systems for immune cell targeting can be extremely interesting. Not only because a more specific delivery can be achieved, but also because the cellular internalization of the targeted nanosystem can be modulated and potentiated. Additionally, the attachment of PRRs ligands on the surface of nanocarriers may boost their immunogenicity, which can be an outstanding strategy for the development of vaccines, since it allows the incorporation of an antigen and the “danger signal” in the same platform (Silva et al., 2013).

### Nanocarriers for imaging approaches

The importance of a deeper knowledge of the dynamic cancer immunological processes has long been realized. The study of these processes *in vivo*, with living cells and the whole organism, is essential to answer this issue more accurately. Cancer disease processes will be better understood and thus improved therapies can surely be developed. For the visualization of these biological dynamic processes *in vivo*, methods have to provide a real-time *in situ* fast response, as well as be non-invasive and with high sensitivity and stability (Wang et al., 2013b).

The use of targeted nanoplatforms for this purpose enables a more specific interaction with the intended target, with minimal interference to the biological system (Ballou et al., 2004). Additionally, nanocarriers may be functionalized with single or multiple ligands, which may be important for the design of complex experiments. The targeting of ligands may enhance the selective recognition of the nanodelivery systems by cells, facilitating their endocytosis. This will allow nanosystems to be used as non-invasive localization, monitoring and assessment platforms, for instance, for site-specific intracellular characterizations and real-time tracking (Ruan et al., 2007).

#### Fluorescence imaging techniques

Fluorescence imaging is an optical imaging method based on the excitation/emission of molecules (Cai and Chen, 2007). The use of fluorescent molecular probes—as fluorescent dyes and fluorescent proteins—has been widely applied in the labeling of biomolecules, cells and tissues. Although these probes are already used *in vivo*, for instance in retinal angiography and visualization of arteries, they are unsuitable for real-time imaging assays, regarding their low photostability and sensitivity at the cellular and molecular levels (Santra and Malhotra, 2011). The application of fluorophores in real-time *in vivo* imaging has also been limited by the high absorption of optical signal by tissues and body fluids in the UV and visible wavelength. The light scattering caused by tissues that attenuate the optical signal and the tissue auto-fluorescence that influences the background signal is also a limitation (Santra and Malhotra, 2011). Additionally, some fluorescent probes may be toxic for cells and body (Li et al., 2013). Several NP-based strategies have been proposed to overcome the limitations of fluorescent dyes for real-time *in vivo* imaging (Supplementary Material) (Santra and Malhotra, 2011; Wang et al., 2013b).

Fluorescent-labeled NPs are more stable in the body and increase the detection sensitivity and photostability. In the same platforms, a great number of probe molecules can be incorporated, in opposite to a single conventional molecule. Also, in NPs, fluorescent dyes can be protected from quenching and degradation (Santra and Malhotra, 2011; Wang et al., 2013b).

The most extensively studied nanosystems for fluorescence imaging are quantum dots (QDs) (Cai and Chen, 2007), inorganic fluorescent NPs that can be based on metallic or semiconductor materials, such as CdSe and CdTe (Ballou et al., 2004). As reviewed by Cai and Chen, in ideal conditions, QDs can have better properties than organic fluorescence probes. These include high resistance to degradation and photobleaching, high quantum yields, high molar extinction coefficients, continuous absorption spectra covering from UV to near-infrared, long fluorescence lifetimes (>10 ns), narrow emission spectra and very long effective Stokes shifts (Cai and Chen, 2007). QDs have been used for innumerous applications, from cell tracking (Voura et al., 2004) to mapping of sentinel lymph nodes (Ballou et al., 2007). QDs can be used to identify several ligands in the same experiment, using multiple colors and intensities to detect different structures (Ballou et al., 2004). The potential use of DC-targeting QDs as both fluorescent NPs for *in vivo* and *in vitro* imaging, and antigen-delivery system has also been investigated. In this study, it was proved that QDs display promising properties for combined priming and immunoimaging of DC (Sen et al., 2008). Functionalization and modifications of the surface of QDs with PEG chains and ligands for active targeting, such as peptides and antibodies, have been under research to improve the application of these nanosystems in the biomedical field (Ballou et al., 2007; Cai and Chen, 2007). QD conjugates are already commercially available for immunospecific labeling (Ballou et al., 2004). Thus, the development of multifunctional nanoplatforms holds a great promise for the future of biomedicine, since it will be possible to combine simultaneously both diagnosis and therapy in the same nanostructure (Kim et al., 2008a).

Several other groups have suggested the use of silica-based NPs (siNPs) as an interesting strategy to perform imaging assays using fluorescence (Santra et al., 2005; Kim et al., 2008a; Wang et al., 2013b). siNPs have been used for high sensitive and specific *in situ* labeling and tracking of cell surface receptors (He et al., 2004, 2007). Relying on the affinity of antigen-antibody or ligand-receptor interactions, NPs were functionalized with antibodies and ligands and applied as an immunediagnostic method (He et al., 2002). siNPs have also been used as a non-invasive tool for intracellular labeling, tracking and sensing in living cells, contributing with novel information about dynamic biological processes of subcellular structures, such as lysosomes and endosomes (Shi et al., 2010). Finally, siNPs were applied to better understand the biodistribution and fate of NPs, *in vivo* (Wang et al., 2013b).

#### Molecular imaging techniques

The key role of immune cells in the development of future immunotherapeutic approaches against chronic pathologies, mainly cancer diseases, has fostered the design and optimization of different real-time imaging techniques, avoiding the classic *ex vivo* histologic analysis (Kircher et al., 2011; Ahrens and Bulte, 2013; Liu and Li, 2014). In fact, most of the information obtained for immune cell tracking has arisen from optical and confocal microscopy and flow cytometry. Two-photon microscopy allowed the observation of different immune cells in their biological environment at real time (Progatzky et al., 2013). However, despite being a powerful tool to observe these highly motile cells and characterize their interaction with native environment, this imaging technique is unsuitable for detection of deeper events due to tissue opacity (Dzhagalov et al., 2012).

Bioluminescence imaging techniques, on the other hand, enable deeper tissue penetrations while tracking immune cells *in vivo*. Even though, it is one of the most commonly used techniques for immune cell tracking *in vivo*, allowing whole-body non-invasive tomography. This technique is only useful for preclinical studies in small animals, due to the limits related to the attenuation of light in tissues (Kircher et al., 2011).

All near-infrared (NIR) multiphoton microscopy methods are potential techniques for deep tissue imaging but further studies are needed to better characterize the capabilities of these NIR-excitation techniques and background reduction (Joshi et al., 2013).

Magnetic resonance imaging (MRI), ultrasound, positron emission tomography (PET) (Yaghoubi et al., 2009), single photon emission tomography (SPECT) and X-ray computed tomography (CT) are the imaging techniques approved for medical applications (Bernsen et al., 2014). PET and SPECT are high-sensitivity and low-resolution techniques, while MRI and CT provide high-resolution images (Liu and Li, 2014). However, the use of radionuclide-based techniques, as PET and SPECT, has brought questions regarding their safety (Laskey et al., 2010). In addition, their combination with additional methods is fundamental to obtain an anatomical image. Therefore, the combination of these different imaging modalities constitutes a multimodality imaging method that has been explored in preclinical and clinical development, including SPECT/CT and MRI/PET (Naumova et al., 2014).

Among these techniques, MRI is the most versatile and sensitive method allowing the study of immune cell morphology and function (Ahrens and Bulte, 2013). In fact, innovative and safer techniques are emerging from the use of different biocompatible cell labeling probes and MRI to obtain high-resolution images without using ionizing radiation (Sosnovik and Nahrendorf, 2012; Thu et al., 2012). The signal used for MRI arises from the water protons (1H) or different fluorinated molecules (e.g., ^19^F) under a static magnetic field and after pulsed by a radio-frequency radiation, which alters the equilibrium of their nuclei. The MRI signal will then result from a transient voltage determined by the properties of labeled tissue (Ahrens and Bulte, 2013).

This non-invasive and safe imaging technique has been expected to track immune cells *in vivo*, enabling the characterization of their biodistribution and fate. MRI also seems suitable for the detection/quantification of surface markers and secreted factors resultant from biological processes occurred *in vivo* at a particular disease stage (Lu et al., 2013; Naumova et al., 2014). The rapid evolution in this field, advanced by the potential efficacy of next-generation cellular-based therapeutic approaches (e.g., immunotherapy and stem cell-based therapy), will certainly make this method a crucial tool to follow detailed biological and immunological processes *in vivo*.

The successful application of these *in vivo* cell-tracking tools can potentially optimize image-guided diagnostics and the overall efficacy of different therapeutic options. Particularly, those based on the modulation of endogenous cells support the selection of a specific treatment, the choice of the best administration route and also the use of a correct dose for each patient (Ahrens and Bulte, 2013).

Different exogenous cell-labeling probes have been explored but superparamagnetic iron oxide (SPIO) nanoparticles and perfluorocarbon (PFC) nanoemulsions seem to be the most promising for those advanced MRI-based techniques (Supplementary Material). Moreover, these are the unique *in vivo* MRI cell-labeling techniques approved for human clinical trials, and thus will be further discussed (Ahrens and Bulte, 2013).

***Nano-based systems for MRI real-time tracking of immune cells***. Different nanosystems (Supplementary Material) have been developed for MRI-based *in vivo* cell tracking, but the negative contrast agents based on SPIO and PFC constitute the most explored ways to control MRI signal and consequent detection (Hawrylak et al., 1993; Bulte and Kraitchman, 2004). SPIO contrast agents are small particles composed by ferrous and ferric oxides, usually coated by dextran. Even though, these ionic NPs have been modified by other biodegradable polymer (e.g., chitosan, PEG, siloxanes, polyaniline, glyceryl monooleate) and labeled with targeting moieties to potentiate their delivery to certain tissues (Supplementary Material) (Shubayev et al., 2009; Dilnawaz et al., 2010). These MRI-based contrast agents strongly perturb the magnetic field of the region in which they are embedded. The water molecules will sense that alteration in the magnetic field and the resultant loss of signal will lead to a dark image (Ahrens and Bulte, 2013). On the other hand, fluorinated-based probes directly label targeted cells and thus the MRI signal is dependent on the number of fluorine atoms and labeled cells, which can be observed in their biological environment (Srinivas et al., 2012).

The labeling of cells using these nano-based systems can be performed *ex vivo* or *in vivo*, through their direct administration in the body. The labeling of immune cells *ex vivo* with SPIO NPs has been explored to track and clarify migratory patterns of diverse immune cells, as NK (Daldrup-Link et al., 2005), cells from T lineage (Kircher et al., 2011), and DCs (De Vries et al., 2005; Rohani et al., 2011) used during immunotherapeutic cancer approaches. Innovative immunotheranostic strategies under development combine these metal ion-based NP with targeted nanoparticulate cancer vaccines. One interesting study has shown multifunctional iron oxide NPs formulated in order to deliver carcinoembryonic antigens to DCs and be detected by MRI (Cho et al., 2011). Alternatively, some SPIO NPs have been developed to label DCs membranes by modifying their surface with CD11c antibodies, promoting receptor-mediated endocytosis (Ahrens et al., 2003; Yu et al., 2012). Despite being a promising approach against cancer disease, their clinical translation is still unclear.

The *ex vivo* labeling of a DC-based cancer vaccine by SPIO NP was used in the first clinical trial that involved the cell tracking by MRI techniques, where it was possible to detect the target lymph node only in half of the patients with melanoma (De Vries et al., 2005).

T cells have been sorted and cultured with SPIO NPs, mostly coated by transfection agents, as poly-L-lysine or protamine sulfate, to promote their capture due to the non-phagocytic nature of these immune cells (Arbab et al., 2005; Thorek and Tsourkas, 2008; Thu et al., 2012). These intracellular labeling was also attempted through the use cell-penetrating peptides and HIV-TAT (Torchilin, 2008).

The *in vivo* labeling of immune cells by SPIO NPs is often used to track monocytes and macrophages to characterize inflammatory events, due to their phagocytic behavior (Settles et al., 2011). The *in vivo* labeling can be achieved by the intravenous administration of SPIO NPs, or alternatively after their direct injection into tumor tissue. Both options were successfully used to label immune cells and track their migration pattern toward lymph nodes, which allows for example the definition of tumor specific stage (Harisinghani et al., 2003).

It is important to emphasize that the cell labeling strategy must not alter the function and normal phenotype of immune cells, which could limit the efficacy of cellular-based therapies. The SPIO NPs are known as safe systems due to their biodegradability nature and usual rapid metabolization *in vivo* (Yu et al., 2012). Therefore, the SPIO-based cell labeling is mostly suitable for short-term studies. On the other hand, false positives may be detected after the accumulation of the detection agent in macrophages after the destruction of labeled cells (Ahrens et al., 2003; Thorek and Tsourkas, 2008). This disadvantage is in fact common to different imaging reagent-labeled techniques.

The ^19^F MRI is a highly sensitive technique that allows the direct quantification of labeled immune cells, as T cells and phagocytic cells, either *in vivo* or *ex vivo* (Srinivas et al., 2009; Helfer et al., 2010). Unlike SPIO NPs, this labeling method usually does not detect false positives and, once is not metabolized *in vivo*, constitutes a suitable approach for long-term studies (Janjic and Ahrens, 2009; Srinivas et al., 2012).

The droplet surface of these PFC colloidal systems has been changed with charged entities to potentiate their efficient delivery at intracellular level. Therefore, the safety of these labeling systems is increased, which has been shown using different immune cells, as DCs and T cells (Ahrens et al., 2005; Srinivas et al., 2009; Helfer et al., 2010; Ahrens and Bulte, 2013).

Recent studies have shown the promising combination of ^19^F labeling techniques with fluorescence or NIR probes, as well as with nuclear magnetic resonance (NMR) (Patel et al., 2013). Even though, the use of these colloidal system for cell tracking is considerably recent and further studies are urged in order to confirm these indications.

## Animal models for the translation of immunotherapeutic approaches

The successful translation of alternative immune-based approaches for cancer therapy into the clinic is highly dependent on the development of preclinical animal models that adequately mimic human disease progression. Several models have been developed and successfully used to study cancer mechanisms of disease and the efficacy of conventional therapeutic options (Budhu et al., 2014).

Accordingly, models currently used to evaluate therapeutic antitumor efficacy at preclinical level are based on transgenic systems and the transplantation of *in vitro* grown cancer cells into healthy animals or in humanized mouse models—human tumor xenograft models (Ostrand-Rosenberg, 2004). The implantation of human cell lines dictates the use of immunocompromised mice –T-cell deficient—to allow the establishment of cancer disease. Besides being one of the most used models to study cancer disease and the effect of cytotoxic therapies, those are definitely not suitable to test the efficacy of immunotherapeutic strategies as it is not possible to evaluate the effect of adaptive immune response in tumor development (Legrand et al., 2009). However, different approaches are currently being explored to improve their application toward the reconstitution of the immune system using human cells (Carpenito et al., 2009; Legrand et al., 2009; Pedroza-Gonzalez et al., 2011). Still, the evaluation of the outcome of different immunotherapeutic options has been possible due to development of different mouse cancer cell lines, which can be further modified if needed: B16 melanoma, CT26 colon carcinoma, TRAMP (transgenic adenocarcinoma of the mouse prostate model) prostate cancer, 4T1 breast cancer, EL4 T lymphoma (Greenberg et al., 1995). Even tough, there is usually a rapid tumor growth after the subcutaneous administration of those cells and therefore these models do not mimic the long-lasting host-tumor interactions resultant from the spontaneous implementation of this disease. On the other hand, the transplantable tumors are very versatile for prophylactic studies as it allows establishment of different vaccination settings, allowing an immune response before the induction of cancer disease and consequent immunosuppressive outcomes.

The spontaneous and multi-step tumor development, including the cross-talk between cells within tumor microenvironment is possible in genetic modified animals (Dougan et al., 2011). However, these animals need to be evaluated for longer periods of time. In addition, the presence of mutations in a permanent manner, in contrast to what happens in cancer disease, has been associated with higher variability and tolerance and consequently, lower effectiveness of different immunotherapeutic options (Hurwitz et al., 2000; Ercolini et al., 2005).

As a result, there is an urgent need for animal models recapitulating cancer disease, and all results should be discussed having in consideration animal model specificities and limitations. In addition, different types of animal models should be tested in order to better characterize the obtained antitumor evidences for clinical translation.

## Conclusions and future perspectives

Despite the improvement observed in chemotherapy and radiotherapy for cancer treatment, the battle against this disease seems to have more chances to be achieved through the combination of different therapeutic modalities. Immunotherapeutic approaches have emerging as promising tools to address the heterogeneity of this disease, namely those immune cell mediated cancer therapies. It is possible to underline the advances obtained with the approval of anti-CTL4 monoclonal antibody by the FDA, and great expectations have arisen from the use of different approaches to modulate the function of immune cells within tumor site. Among those strategies, the outcome of cancer vaccines can be highlighted. To monitor and guide the development of cellular therapies and the *in situ* manipulation of immune cells, the improvement of non-invasive imaging strategies to obtain detailed information regarding the biological processes within the complex tumor microenvironment is imperative. We foresee the use of non-toxic nanotechnology-based systems able to combine the specific (i) targeting of immune cells, promoting the controlled delivery of different molecular entities to modulate the cell-cell interactions; and (ii) tracking through the inclusion of different probes to improve safety, specificity and sensitivity of cell-labeling methods and imaging approaches. These immunotheranostics are expected to enable a rational definition of treatment plans for a particular patient, resulting in better clinical outcomes and enhanced control of the disease, which can also promote their translation into marketed systems.

### Conflict of interest statement

The authors declare that the research was conducted in the absence of any commercial or financial relationships that could be construed as a potential conflict of interest.
