# Supplementary material for: Cancer immunotherapy: nanodelivery approaches for immune cell targeting and tracking
Source: Front Chem. 2014 Nov 26;2:105. doi: 10.3389/fchem.2014.00105 (PMC4244808; doi:10.3389/fchem.2014.00105)
Supplement: Supplementary file 1 [file Presentation1.PDF]

Cancer immunotherapy: nanodelivery approaches for immune cell

Supplementary Material

Table 1 – Examples of nanotechnology-based systems used for targeted imaging and/therapy using imaging approaches

TRACKING & TARGETING			
IMAGING TOOL	NANOSYSTEMS/COMPOSITION	APPLICATION/POTENTIAL	REFERENCES
Fluorescence	Non-targeted Quantum Dots (QDs)	B16 Melanoma cell tracking	(Voura et al., 2004)
	Prostate specific membrane antigen (PSMA)antibody-labeled QDs	Targeting and imaging prostate cancer *in vivo* (mice)	(Zhang et al., 2013)
	Alpha-fetoprotein (AFP) antibody-QDs conjugates	Detection of this hepatocellular carcinoma marker in serum	(Yu et al., 2007)
	Anti-HER2 scFv-QDs-immunolipossomes	*In vivo* breast cancer cell imaging	(Weng et al., 2008)
	PEG-cd chalcogenide based QDs	Breast cancer cell imaging	(Poulose et al., 2012)
	Carbohydrates (D-mannose, D-galactose, or D-galactosamine)-capped-PEG QDs	*In vitro* imaging and *in vivo* liver targeting	(Kikkeri et al., 2009)
Fluorescence/bioluminescence	Antibody-labeled luminescent QDs	Tumor-targeting and imaging	(Gao et al., 2004)
Multiphoton fluorescence	Non-targeted cadmium (Cd) selenide (Se)-zinc (Zn) sulfide QDs	Vasculature imaging	(Larson et al., 2003)
NIRF	Non-targeted CdTe/CdSe core/shell QDs	Sentinel lymph-node mapping	(Kim et al., 2004;Tanaka et al., 2006)
	anti-Her2 monoclonal antibody (Ab)-conjugated CdTe/CDSe/ZnSe QDs-labeled	Her2-cancer cells tracking	(Rizvi et al., 2014)

	Indium&arsenic-QDs	Sentinel lymph-node mapping	(Kim et al., 2005)
	Arginine-glycine-aspartic acid (RGD)-peptide labeled QDs	*In vivo* tumor cells and vasculature imaging (mice)	(Cai et al., 2006)
	RED peptide-QDs	Targeting and imaging αvβ3-overexpressed cancer cells *in vivo*	(Cai et al., 2006)
CT	Bismute sulphide (Bi_2S_3)-based NPs	Vasculature imaging	(Rabin et al., 2006)
Ultrasonograph y	αvβ-targeted PFC NPs	Tumor neovasculature detection	(Hughes et al., 2006)
Raman imaging	RGD-labelled Single-walled carbon nanotubes (SWNTs)	Tumor tracking and imaging *in vivo*	(Zavaleta et al., 2008)
PET	anti-CD105 (TRC105)-conjugated ^{64}Cu-nanographene oxide	*In vitro*, *ex vivo* and *in vivo* tumor neovasculature targeting and imaging	(Hong et al., 2012)
MRI	Ferumoxides (dextran-coated SPION)	Clinically approved (Feridex IV Berlex Laboratories; and Endorem, Guerbet) in USA, Europe, and Japan for liver imaging	(Wang et al., 2001)
	Ferucarbotran (carboxydextrane-coated SPIO)	Clinically approved (Resovist, Bayer Healthcare) in Europe and Japan for liver imaging	(Reimer and Balzer, 2003)
	Ligand-labeled SPIONs	Tracking of dendritic cells (DCs), cytotoxic T cells, natural killer (NK) cells	(Ahrens et al., 2003;Kircher et al., 2003;Daldrup-Link et al., 2005)
	Ovalbumin-specific splenocytes labeled with SPIO NPs	*In vivo* tracking and imaging	(Smirnov et al., 2006)

	Cationic coated-ferumoxides NPs	Non-phagocytic cell tracking (e.g. stem cells)	(Frank et al., 2003;Arbab et al., 2004)
	Manganese oxide (MnO) or SPIO NPs electroporation	*In vitro* and *in vivo* glioma cell imaging	(Gilad et al., 2008)
	Magnetodendrimers	*In vivo* tracking of mesenchymal stem cells (MSCs) and human neural stem cells (NSCs)	(Bulte et al., 2001)
	SPIO NPs	Neural progenitor cells, bone marrow stromal cells, and MSCs in glioma models	(Zhang et al., 2004)
	Ferumoxide-protamine sulfate complex (Fe-Pro) labeled NSCs	*In vivo* cell tracking in glioma model	(Thu et al., 2009)
	Fe-Pro labeled CD34+/AC133+ endothelial progenitor cells (EPCs)	Neovasculature imaging	(Arbab et al., 2006)
	Negatively charged fluorescent SPIONs and positively charged peptides complexes	*In vitro* and *in vivo* stem cell tracking	(Lee et al., 2009b)
	Perfluorocarbon (PFC) NPs	*In vivo* stem cell tracking	(Partlow et al., 2007)
	β-Glucan-coated SPIONs	Immune cell targeting within liver metastasis site for diagnosing metastasis in a metastatic mouse liver model	(Vu-Quang et al., 2012)
NIRF and PET	1,4,7,10-tetraazacyclodocecane-N, N’, N’’, N’’’-tetraacetic acid (DOTA)-QDs-RGD peptide	Tumor-targeting efficacy	(Cai et al., 2007)

MRI/Fluorescence	Gadolinium (Gd)-containing polymer NPs	Macrophage imaging	(Hashim et al., 2014)
MRI/NIRF	(Cy5)BSA-superparamagnetic iron oxide NPs (SPION) - anti-plectin-1 monoclonal antibody	Pancreatic ductal adenocarcinoma imaging	(Wang et al., 2014)
PET/MRI	^{18}F-dextran-coated SPIONs	*In vivo* targeting and imaging of lymph nodes (≅1mm)/cancer metastasis	(Devaraj et al., 2009)
PET/MRI	64Cu-DOTA-labelled IONPs	Vascular inflammation imaging	(Jarrett et al., 2008)
PET/NIRF/MRI	64Cu-DOTA-IONPs- Cy5.5	*In vivo* tumor imaging	(Xie et al., 2010)
PET/RAMAN	PEG-DOTA-SWNTs-RGD	*Ex vivo* tumor imaging	(Liu et al., 2007)
SPECT/MRI	^{99m}Tc-IONPs	*In vivo* breast cancer and malignant melanoma targeting and imaging	(Madru et al., 2012)
**TARGETING/IMAGING & THERAPY**			
IMAGING TOOL	NANOSYSTEMS/COMPOSITION	APPLICATION/POTENTIAL	REFERENCES
Optical imaging	Antibody-drug conjugate — Cetuximab-labeled PEG- AuNPs capped by a Raman reporter (smart SERS gold nanoantennas)	*In vivo* tumor recognition and inhibition	(Conde et al., 2014)
SPECT	^{188}Rhenium (Re)-labelled IONPs	*In vivo* targeting/imaging and therapy of liver cancer cells.	(Liang et al., 2007)
	111-Indium (In) chimeric L6 antibody-PEG-dextran-coated IONPs	Breast cancer tumor targeting/ imaging and alternating magnetic field (AMF) treatment	(Ivkov et al., 2005;DeNardo et al., 2007)

	^{99m}Tc-labeled cobalt ferrite NPs stabilized with ethyl 12-(hydroxyamino)-12-oxododecanoate , poly(lactic-co-glycolic acid) and bovine serum albumin (BSA)	Spleen and liver targeting and thermal therapy	(Psimadas et al., 2012)
SPECT/CT	^{125}I-folate-Au nanorods	*In vitro* and *in vivo* imaging of folate-receptor positive tumors and thermal-ablation therapy	(Jang et al., 2012)
	^{99m}Tc-labeled AuNPs	Prostate and breast cancer imaging and thermo ablative therapy	(Morales-Avila et al., 2011;Ocampo-Garcia et al., 2011;Morales-Avila et al., 2012)
	Tat(49-57)-Lys3-bombesin-conjugated ^{177}Lu/^{99m}Tc-AuNPs	Prostate and breast cancer imaging and radio and thermo-ablative therapy	(Madru et al., 2012)
PET/CT	*64*Cu-DOTA labeled gold nanoshells conjugated to cRGDfK (cyclo-Arg-Gly-Asp-Phe-Lys)	*In vivo* tumor vasculature imaging and tumor necrosis by subablative thermal therapy	(Xie et al., 2011)
Micro-PET/CT	^{64}CuS NPs	*In vitro* and *in vivo* targeting/imaging and Photothermal ablation (NIR light)	(Zhou et al., 2010)
PET/MRI	HIV-1 Tat/fluorescein isothiocyanate/^{68}Ga-labeled IONPs	*In vivo* liver cell tracking and thermo-ablative therapy	(Stelter et al., 2010)

Fluorescenc e	Delivery of MDR-1 siRNA and doxorubicin (DOX) by CdSe/ZnSe QDs modified with β-Cyclodextrin coupled to L-Arg or L-His	Real-time tracking treatment (chemotherapy and gene therapy)	(Li et al., 2012)
	SA coated manganese-doped)NPs Iodified by RGD peptide and RNA.	High delivery efficiency into cancer cells and gene therapy	(Lee et al., 2009a)
	p53 DNA-loaded polyethylenimine (PEI) SPION	Tumor imaging and gene therapy	(Lee et al., 2012)
	Trastuzumab-conjugated Mn-doped magnetism-engineered iron oxide (MnMEIO)	HER2-positive tumor imaging and targeted therapy	(Lee et al., 2007)
	Pluronic F127 stabilized- oleic acid coated SPIONs	Imaging and chemotherapy	(Jain et al., 2009)
	PEG-poly(glutamic acid)-micelles loaded with anticancer drug DACHPt and Gd3+.	Pancreatic tumor accumulation and chemotherapy	(Kaida et al., 2010)
MRI	DOX and Gd-DOTA -loaded lipossomes	Non-invasive *in vivo* monitoring and chemotherapy in Kaposi's sarcoma and B16 Melanoma murine models	(Grange et al., 2010)
	Gd3+ and DOX-loaded Thermosensitive lipossomes (HaT: Hyperthermia activated cytoToxic)	Chemo/hyperthermia therapy on mammary carcinoma	(Tagami et al., 2011)
	Her-2-antibody-labeled DOX-loaded PLGA incorporated hydrophobic MnFe2O4 magnetic nanocrystals (magneto-polymeric nanohybrids)	Breast cancer imaging and chemotherapy	(Yang et al., 2007)
	Ultrasmall superparamagnetic iron oxide (USPIO) NPs loaded into squalenoyl-gemcitabine conjugate (USPIO/SQgem NPs)	*In vivo* chemotherapy and MRI in murine leukemia model	(Couvreur et al., 2006;Desmaele et al., 2012)

	DOX-SPIO-loaded cRGD-labeled PLA micelles	*In vivo* monitoring and chemotherapy in lung carcinoma model	(Blanco et al., 2009)
	Peptide-labeled IONPs	Imaging and tumor neo-angiogenesis treatment through occlusion of tumor vessels by IONPs	(Park et al., 2009;Agemy et al., 2010)
	Photofrin-IONPs entrapped-multifunctional polyacrylamide- based NPs	Monitoring and photodynamic therapy of brain tumors through the photo- activable hemathophorphyrin agent	(Kopelman et al., 2005;Reddy et al., 2006)
MRI/fluorescence	Gd3+-conjugated lipids and AlexaFluor®-labeled siRNA molecules entrapped in PEG-liposomes	Gene therapy, *in vivo* imaging in ovarian murine model and *ex vivo* intracellular trafficking	(Kenny et al., 2011)
MRI/optical imaging	IONPs-Cy7 dye- prednisolone acetate valeranate-loaded nanoemulsion	*In vivo* imaging and therapeutic effect in colon cancer murine model	(Gianella et al., 2011)
MRI/optical imaging	PGN635 antibody-labelled lipossomes entrapping SPIONs	*In vitro* and *in vivo* breast cancer vasculature imaging and chemotherapy	(Zhang et al., 2014)
	DOX-loaded folate-PEG-PLGA NPs entrapped with IONPs and CdSe/ZnS QDs	Cancer targeted magnetic resonance or optical imaging and magnetically guided chemotherapy	(Kim et al., 2008b)
